# Counterweight mass influences single-leg cycling biomechanics

**DOI:** 10.1371/journal.pone.0304136

**Published:** 2024-06-07

**Authors:** Michael J. Asmussen, Erica Casto E., Martin J. MacInnis, Benno M. Nigg

**Affiliations:** 1 Department of Kinesiology, Faculty of Education, Vancouver Island University, Nanaimo, BC, Canada; 2 Faculty of Kinesiology, University of Calgary, Calgary, AB, Canada; 3 Department of Kinesiology, University of Massachusetts-Amherst, Amherst, MA, United States of America; Polytechnic University of Marche: Universita Politecnica delle Marche, ITALY

## Abstract

**Introduction:**

Single-leg cycling is a commonly used intervention in exercise physiology that has applications in exercise training and rehabilitation. The addition of a counterweight to the contralateral pedal helps single-leg cycling mimic cycling patterns of double-leg cycling. To date, no research has tested (a) the influence of a wide range of counterweight masses on a person’s cycling biomechanics and (b) the optimal counterweight mass to emulate double-leg cycling.

**Objectives:**

The purpose of this study was to determine the effects of varying counterweights on the kinematics (joint angles) and kinetics (joint moments, work) of cycling using a 3D analysis.

**Methods:**

Twelve participants cycled at 50W or 100W with different counterweight masses (0 to 30 lbs, 2.5 lbs increments), while we analyzed the pedal force data, joint angles, joint moments, and joint power of the lower limb using 3D motion capture and 3D instrumented pedals to create participant-specific musculoskeletal models.

**Results:**

The results showed that no single-leg cycling condition truly emulated double-leg cycling with respect to all measured variables, namely pedal forces (*p* ≤ 0.05), joint angles (*p* ≤ 0.05), joint moments(*p* ≤ 0.05), and joint powers (*p* ≤ 0.05), but higher counterweights resulted in single-leg cycling that was statistically similar (*p* > 0.05), but descriptively, asymptotically approached the biomechanics of double-leg cycling.

**Conclusion:**

We suggest that a 20-lb counterweight is a conservative estimate of the counterweight required for using single-leg cycling in exercise physiology studies, but further modifications are needed to the cycle ergometer for the biomechanics of single-leg cycling to match those of double-leg cycling.

## 1.0 Introduction

Single-leg cycling is a common form of unilateral exercise that has been used frequently to study various aspects of human physiology [1–4]. As this mode of exercise allows legs to be exercised independently, it reduces the amount of active muscle mass during an exercise intervention by approximately half. Accordingly, the required total mechanical work, metabolic rate and associated cardiorespiratory demands of exercise are much lower for single-leg cycling at the same relative power output (i.e., W/leg) as double-leg cycling, with responses being comparable across modes at the same absolute power output (i.e., total W) [5–7]. The oxidative [8] and perfusive capacities [9] of the quadriceps muscle group exceed the values required during large muscle mass exercise, and because more blood flow (i.e., oxygen delivery) can be directed to the active leg in single-leg cycling [5], each leg can sustain a higher relative power output in various tasks exercised unilaterally as compared to bilaterally [6, 10]. The ability to train legs independently at higher relative power outputs with lower cardiorespiratory stress creates a myriad of possibilities to use single-leg cycling in both physiology and biomechanical studies and different applied settings.

Single-leg cycling has been used in various training and rehabilitation settings. For example, cyclists who performed high-intensity interval training with single-leg cycling exercised at a higher relative power output and demonstrated greater increases in skeletal muscle oxidative capacity compared to cyclists who performed the same training protocol with double-leg cycling [10]. Similarly, individuals with chronic obstructive pulmonary disease (COPD) who trained for 7 weeks with single-leg cycling were able to exercise at a greater relative power output–due to reduced ventilatory demands and dyspnea [11]–and achieved greater increases in double-leg peak oxygen uptake ($\dot{V}O_{2\mathrm{peak}}$) compared to those who trained with double-leg cycling [12]. The ability to train legs independently is also advantageous when one leg has a reduced capacity to train, and single-leg cycling has been used as a rehabilitation strategy after anterior cruciate ligament surgery [13] or meniscectomy and casting [14], allowing individuals to maintain physiological fitness despite having reduced capacities for physical activity due to injury. Even though there are a variety of applications, discussing and/or improving the method of single-leg cycling has received little attention.

Unassisted single-leg cycling is cumbersome, as the ipsilateral hip flexor muscle group need to be active through the upstroke phase of the pedal revolution. A variety of methods have been used to facilitate single-leg cycling to make it more similar to the double-leg cycling counterpart, including pairs of humans cycling the same flywheel [15], springs [16], fixed flywheels [17], and counterweights [10]. Of these methods, the use of a counterweight is a relatively simple adjustment to modify a cycle ergometer, only requiring a mass to be attached to the contralateral crank arm [10]. Compared to unassisted single-leg cycling, the addition of a 10.0–11.6-kg counterweight (i.e., 22–25 lbs) yields similar lower limb kinematics and kinetics [18–20], more similar muscle activation patterns [19], and similar $\dot{V}O_{2}$ and heart rate responses [5] relative to double-leg cycling at the same absolute power output. While counterweighted single-leg cycling with ~25lbs mimics double-leg cycling reasonably well, empirical justification is lacking for choosing the mass of the counterweight.

The purpose of this experiment was to determine the influence of counterweight mass on single-leg cycling 3D kinetics and kinematics. Briefly, participants performed short bouts of single-leg cycling with counterweight masses ranging from 0 to 30lbs (i.e., 0–13.61 kg) in a randomized order at two different power outputs (50W and 100W) on a cycle ergometer instrumented with 3D force pedals and retro-reflective markers. We hypothesized that increasing the mass of the counterweight would improve congruence between single-leg and double-leg cycling to a point after which there would be a negative effect of further increasing the counterweight mass.

## 2.0 Materials and methods

### 2.1 Participants

Based on previous literature in the field [18], the minimum sample size was calculated to be ten participants (minimum effect size: 0.62, α = 0.05, Power (1-β) = 0.80). For this reason, convenience sampling was used to recruit 12 recreational male cyclists (mean ± standard deviation (SD): 27.6 ± 2.8 years, 1.8 ± 0.05 m, 77.3 ± 9.1 kg) who provided their written informed consent to participate in the study, which was approved by the University of Calgary Conjoint Health Research Ethics Board (# REB17-1547). These participants cycled at least once per week and were familiar with using clipless pedals that were used for the experimental trials. Participants were free from any musculoskeletal injuries at the time of the study and recruitment began in January 2018 and finished in August 2018.

### 2.2 Experimental procedures

The participants were required to visit the lab for two sessions. Both sessions involved participants cycling on a magnetically braked cycle ergometer (Velotron, Racermate, Seattle WA). In session 1, participants were familiarized with the experimental protocol, which included double-leg cycling at 100 and 200W (or ~50 and 100W/leg) and single-leg cycling at 50 and 100W. The power output of single-leg cycling was half that of double-leg cycling to match the relative power output requirement per leg between modes. These power outputs were chosen to mimic the range of power outputs that participants would cycle at in a single-leg cycling intervention study and would allow us to assess if single-leg cycling biomechanics are influenced by counterweight mass and the external cycling load. The single-leg cycling involved pedaling with one leg while the contralateral leg rested on a stationary platform adjacent to the cycle ergometer, consistent with previous methods in the field [5, 6]. A custom-machined pedal, designed to hold a range of commercial weights, was affixed to the contralateral crank arm. There were 13 counterweight mass conditions, ranging between 0 and 30 lbs in 2.5-lb increments (i.e., 0, 2.5, 5, 7.5, 10, 12.5, 15, 17.5, 20, 22.5, 25, 27.5, and 30 lbs), and two power outputs conditions (i.e., 50 and 100W), for a total of 26 single-leg cycling conditions. This range of weights was chosen because it should cover the point at which single-leg cycling biomechanics would begin to mimic double-leg cycling and there is sufficient weight resolution to observe this change. There were also limitations beyond a 30lb weight based on pedal design. Cadence was controlled at 80 rpm using visual feedback from the Velotron software and was monitored by the experimenter to ensure the participants maintained this cadence during data collection. Recording of biomechanical data did not occur until the participants could keep the target 80 rpm, and trials were repeated if participants deviated consistently above or below 2 rpm during biomechanical data collection. Participants completed all trials wearing cycling shoes with cleats connected to clipless pedals.

In the session 1, participants performed a subset of the conditions (i.e., 0, 10, 20, and 30 lbs) for approximately 1 minute each to ensure they could cycle proficiently across a range of counterweights. This proficiency was visually evaluated by the experimenter and continued until the participant could move their foot through the pedal revolution with a consistent angular velocity. To prevent any biasing effects due to which leg performed the one-leg cycling condition, half of the participants performed the one-leg cycling with the right leg and the other half of participants performed the cycling with the left leg. This familiarization session took approximately 45 minutes.

Participants began the second session with a 10-min double-leg cycling warm-up at a self-selected power output ranging between 100 and 150W. Participants then performed two-leg cycling for 1-min each at two power outputs (i.e., 100W and 200W), in a randomized order. Subsequently, participants performed the 26 different single-leg cycling conditions consecutively with short breaks between each 1-min cycling bout (as in session 1). Participants rested for a minimum of 30s between cycling bouts and were offered additional rest if required. Participants were instructed that they should not perform the cycling bouts with any excessive fatigue accumulation throughout the experiments to ensure they start each trial in a similar rested state. The order of the counterweight conditions was randomized. Both power output conditions (i.e., 50W and 100W) were performed consecutively within each counterweight condition, and the order of the power output conditions (i.e., 50W first vs. 100W first) was alternated throughout the study across participants. Finally, after completing the 26 one-leg cycling conditions, participants repeated the double-leg cycling conditions that were performed at the beginning of session 2 (i.e., 100W and 200W) but in the opposite order to ensure there were no effects of repeated trials or fatigue on the resulting kinematics and kinetic measures. For each cycling condition, a 3D kinematic and kinetic analysis of the leg that performed the one-leg cycling was conducted.

### 2.3 Kinematics and kinetics

Kinematic data of reflective markers placed on the participants’ lower limbs were collected at 240 Hz using a motion capture system that included eight infrared cameras (Motion Analysis Corporation, Santa Rosa CA, USA). A static trial was collected prior to the start of session 2 with the person on the cycle ergometer. For this static trial, 26 reflective markers (20 mm in diameter) were placed on the participants’ lower limbs and four reflective markers were placed on the pedal. Markers were placed on the pelvis (4, right and left anterior superior iliac spine, right and left posterior superior iliac spine), greater trochanter (1), thigh (4), medial and lateral femoral condyles, shank (4), medial and lateral malleoli, calcaneus (2), 5^th^ metatarsophalangeal joint (1), right toe (1), and 1^st^ metatarsophalangeal joint (1). The pedal markers, the medial malleoli, and the medial condyle were removed during the dynamic cycling.

Kinematic and kinetic data processing and analyses were performed using *OpenSim* [21, 22]. Specifically, participant-specific scaling was performed followed by computation of 3D joint angles using *OpenSim*’s inverse kinematic routine with the proximal segment being the reference segment for the hip, knee, and ankle. Using the kinematic joint angle data, joint angular velocities and joint angular accelerations were derived in a custom Matlab script that used the first and second derivative of the joint angle data to derive the joint velocity and joint acceleration data, respectively, with a central finite difference approximation of derivative method. The kinematic data were filtered using a 2nd-order dual pass Butterworth filter (i.e., zero lag, 4^th^ order method) with a cut off frequency of 15 Hz. 3D joint moments of the hip, knee, and ankle were computed using the kinematic data combined with the force and moment data from instrumented force pedals (Sensix, Poitiers, France). This pedal force data was sampled at 2400Hz using a custom Labview program. These pedals represented the force and moment data in their own coordinate system. Before the dynamic trials, a static trial was performed with markers on the pedals. This procedure allowed us to represent the force and moment data from the pedals in the lab coordinate system to ensure the pedal force was correctly changing based on pedal and crank angle. Using the force and segment kinematic data, along with pedal and crank angle data, internal joint moments were computer using the inverse dynamics routine in *OpenSim* and joint power was computed with a custom Matlab script taking the dot product of joint angular velocity and joint power. With these variables, we examined the joint moments and powers that contribute the most to the cycling pedal stroke, namely hip flexion/extension, knee flexion/extension, and ankle plantarflexion/dorsiflexion for the leg the participant performed the one-leg cycling with. Force data from the pedals were filtered at the same frequency as the kinematic data (i.e., 15 Hz) in accordance with the recommendations by Kristianslund et al. [23]. We examined 30 pedal revolutions per condition.

**Fig 1:**
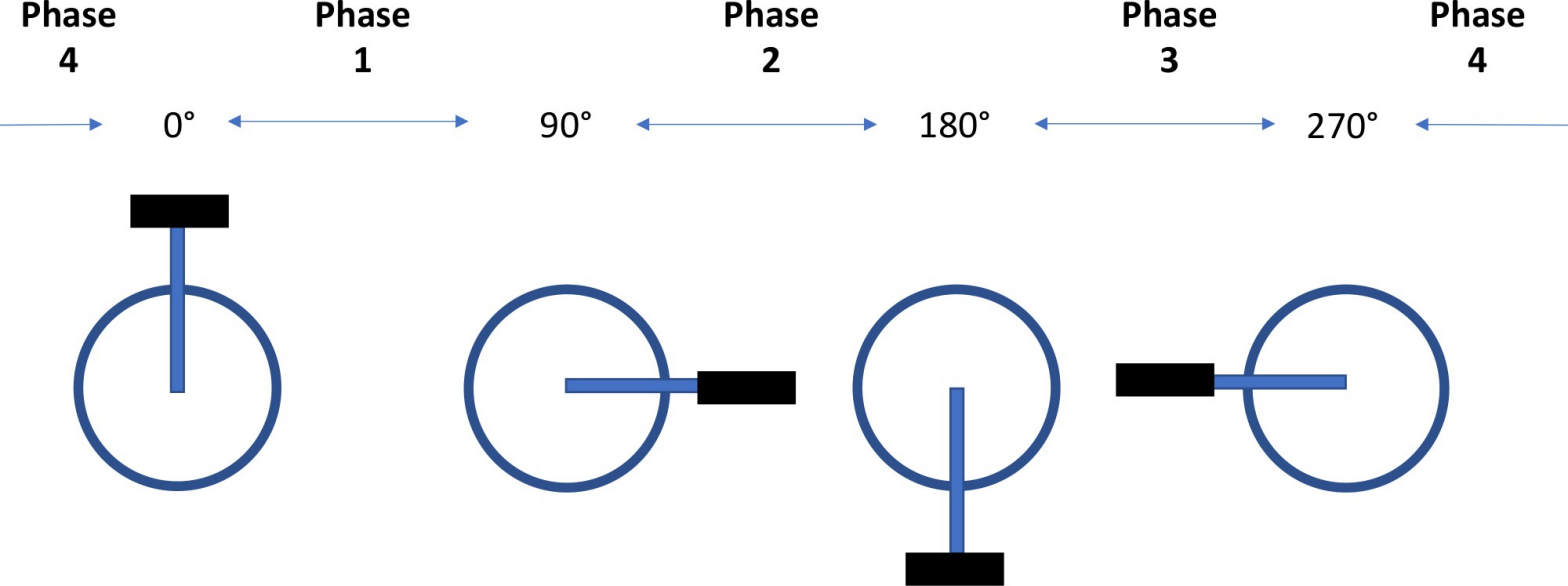
The different phase definitions based on crank angle. Phase 1 occurs from 0°< 90°, Phase 2 occurs from 90°< 180°, Phase 3 occurs from 180°< 270°, and Phase 4 occurs from 270°< to 0°.

To further process the data during the different cycling conditions, we separated the pedal, joint angle, joint moment, and joint power data into four separate phases based on crank angle (Fig 1). Specifically, these phases were: Phase 1 (0°<, top dead-centre to 90°), Phase 2 (90°< to 180°, bottom dead-centre), Phase 3 (180°< to 270°), and Phase 4 (270°< to 360°). During these phases, the peak-to-peak joint angles were computed for each of the four phases. With respect to the pedal data, the peak-to-peak and mean of the forces applied tangential to the crank, parallel to the crank, and in line with the axis of rotation of the crank. Further, the-peak-to peak and mean joint moments were computed as well as positive and negative joint work, defined as the area under the positive and negative joint power curves, respectively, were computed for each phase.

### 2.4 Statistics

A one-way repeated measured ANOVA for cycling condition (14 levels) was performed on each dependent variable within each phase of each power output condition. When the ANOVA results were statistically significant, Dunnett’s test was performed to determine if there were any significant differences in the dependent measures relative to the double-leg cycling condition. The effect size for the individual comparisons for each counterweight mass versus double leg cycling was Hedges’ g. The classification was 0.2 < 0.5, 0.5 = <0.8, 0.8< for a small, medium, and large effect size, respectively. The data were tested for equality of variance using Mauchley’s test of sphericity, and a Greenhouse-Geisser correction was applied to the degrees of freedom when the data did not meet the assumption of sphericity. Separate ANOVAs were performed with each phase of the pedal stroke and each power output because of expected differences in power output and different phases of cycling [24] that were not the focus of this study and not in line with the main research question. Given that no single-leg cycling condition matched the pedal forces observed in the double-leg cycling condition, we performed a linear regression analysis with the peak pedal forces and counterweight mass to predict the mass required for the pedal forces to match double-leg cycling assuming a linear relationship. For descriptive purposes, we also present the crank torque and crank power data throughout a pedal revolution and display the negative and positive work separated into flexion positive and negative work as well as extensor positive and negative work [18].

## 3.0 Results

The results are separated into the joint angle, pedal force, joint moment, and joint work data and further subdivided into the four different phases and two different power outputs. For brevity, reporting statistical data and figures related to the 50/100W power outputs are included in figures in a table form. Furthermore, only significant main effects from the ANOVAs are reported in text, with significant post hoc effects indicated in the relevant figures. The descriptive statistics for each ANOVA and the effect size of the Dunnett’s test are presented in Supplementary files (S1 Fig).

### 3.1 Joint angles

**Fig 2:**
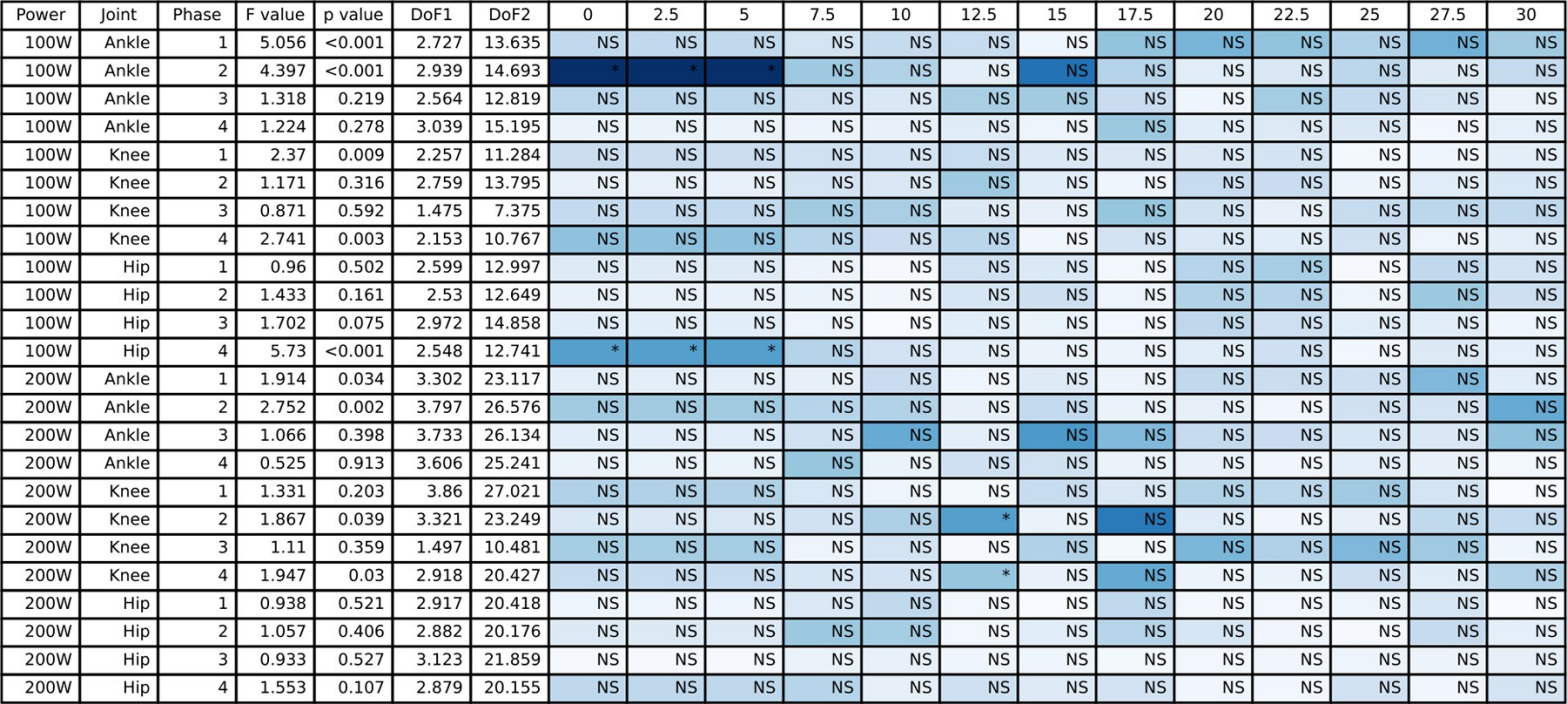
The ANOVA results for the peak-to-peak joint angles and the post hoc Dunnett’s analysis for the different counterweight conditions relative to single-leg cycling. The blue shading represents the effect size with darker blue representing largest effect size for this table and white would indicate an effect size of 0. NS indicates a non-significant post hoc test and an asterisk indicates a significant post hoc test.

**Fig 3:**
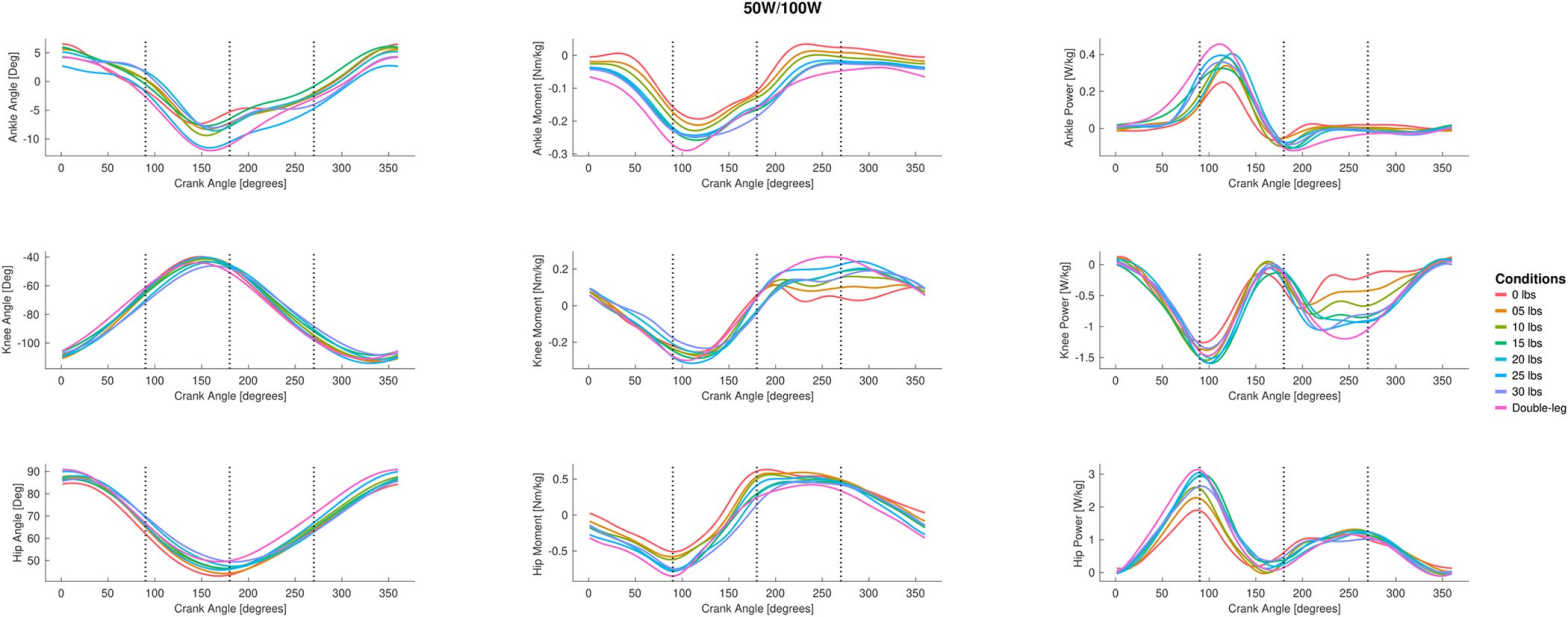
The group mean joint data for different single-leg cycling conditions and double leg cycling across a pedal revolution for 50W single leg cycling (100W double leg cycling). The left column displays the joint angle data for the ankle, knee, and hip, where positive values indicate flexion relative to a neutral posture. The middle column displays the joint moment data for the ankle, knee, and hip with positive values indicating hip flexion, knee extension, and ankle dorsiflexion. The right column displays the joint power data for the ankle, knee, and hip. Data are not displayed for all single-leg cycling counterweight condition for sake of display clarity.

**Fig 4:**
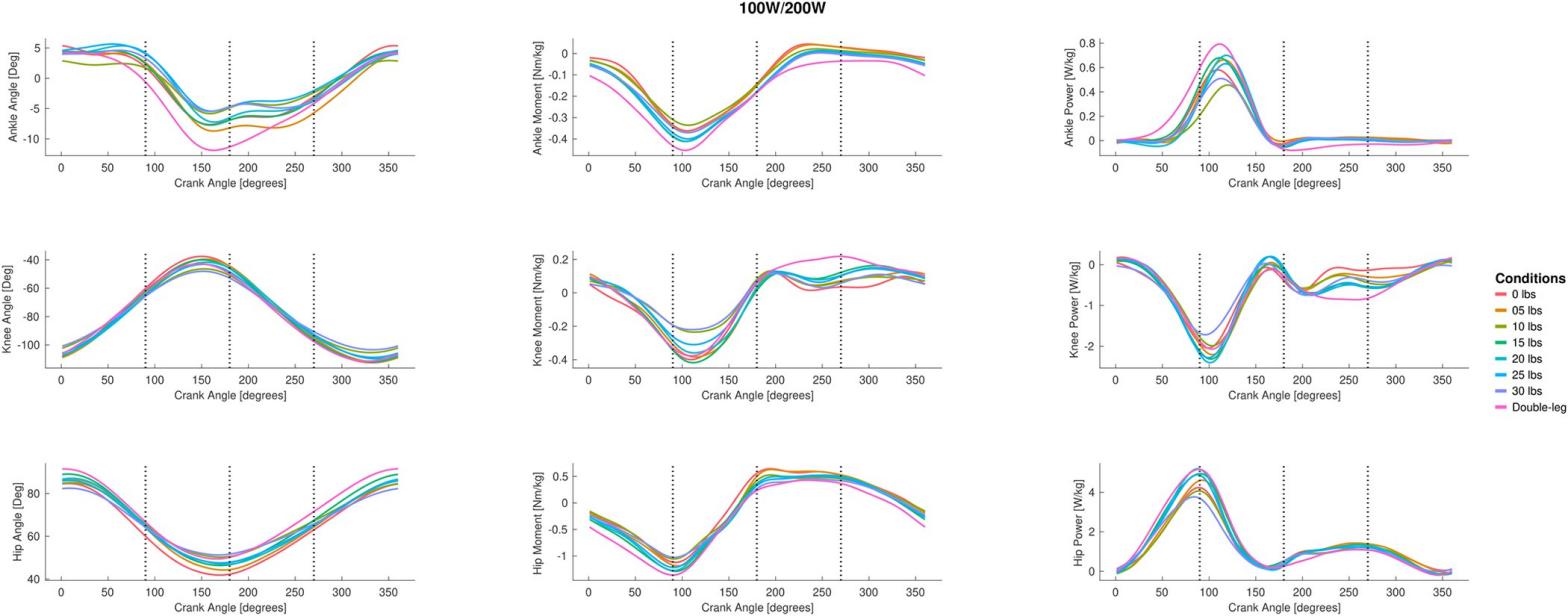
The group mean joint data for different single-leg cycling conditions and double leg cycling across a pedal revolution for 100W single leg cycling (200W double leg cycling). The left column displays the joint angle data for the ankle, knee, and hip. The middle column displays the joint moment data for the ankle, knee, and hip. The right column displays the joint power data for the ankle, knee, and hip. Data are not displayed for all single-leg cycling counterweight conditions for sake of display clarity.

The full joint angle ANOVA with peak-to-peak joint angle results for the hip, knee, and ankle are presented in Fig 2, and the group mean joint angles for the hip, knee, and ankle throughout the pedal stroke are plotted in Fig 3 (50/100 W) and Fig 4 (100/200 W). At 50/100W, there were significantly different peak-to-peak joint angles for the ankle in Phases 1 and 2 only. Peak-to-peak knee joint angles were significantly different in Phase 1 and 4 at 50/100W, but no differences as a result of the post-hoc Dunnett’s test. Peak-to-peak hip joint angles were significantly different at 50/100 W in Phase 4. See Fig 2 for post hoc test results for these significant main effects. There were no significant differences for peak-to-peak joint angles when cycling at the higher 100/200 W power output for the ankle or hip joints during any phase, but the knee joint did show differences for one condition (i.e., 12.5 lbs) in Phase 2 and Phase 4.

### 3.2 Pedal forces

**Fig 5:**
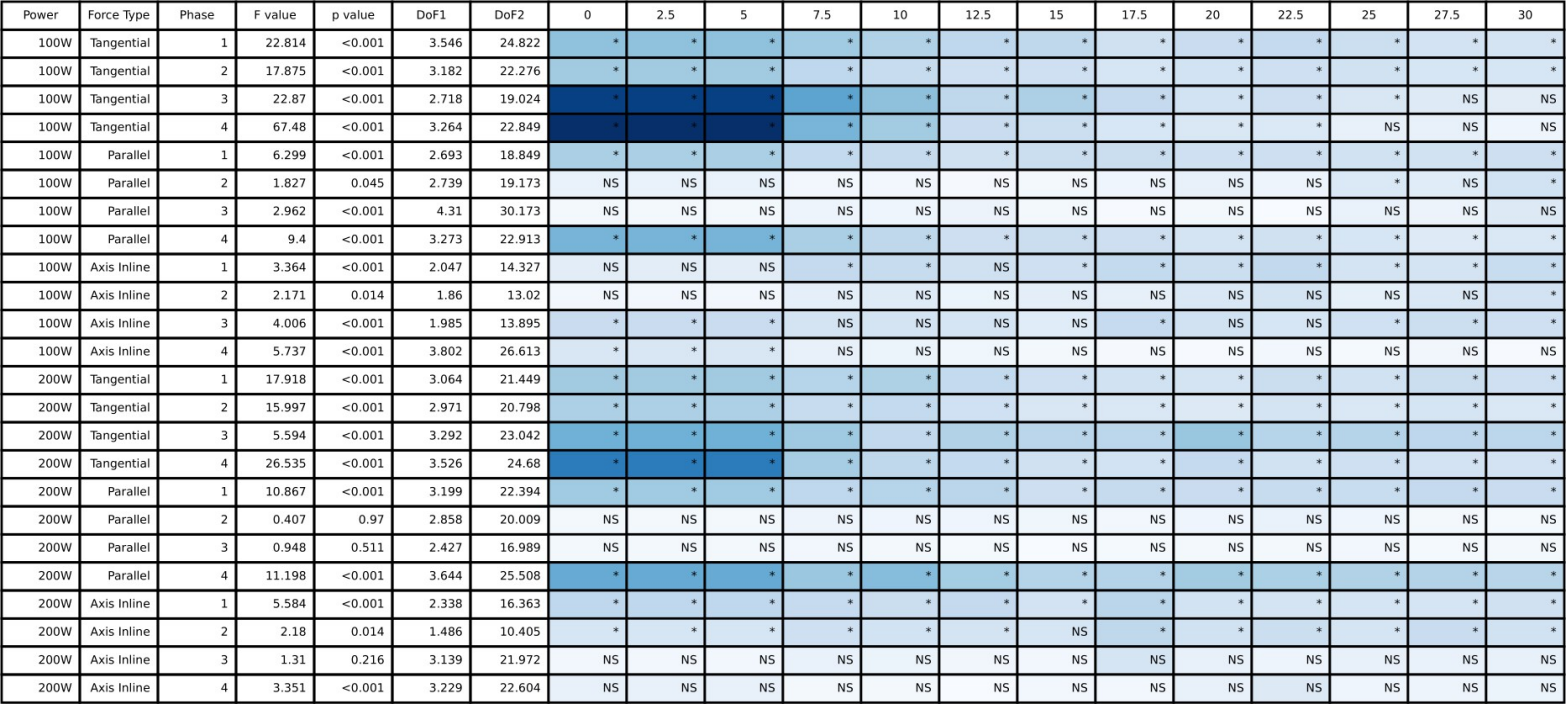
The ANOVA results for the peak-to-peak pedal force data and the post hoc Dunnett’s analysis for the different counterweight conditions relative to double-leg cycling. The blue shading represents the effect size with darker blue representing largest effect size for this table and white would indicate an effect size of 0. NS indicates a non-significant post hoc test and an asterisk indicates a significant post hoc test.

**Fig 6:**
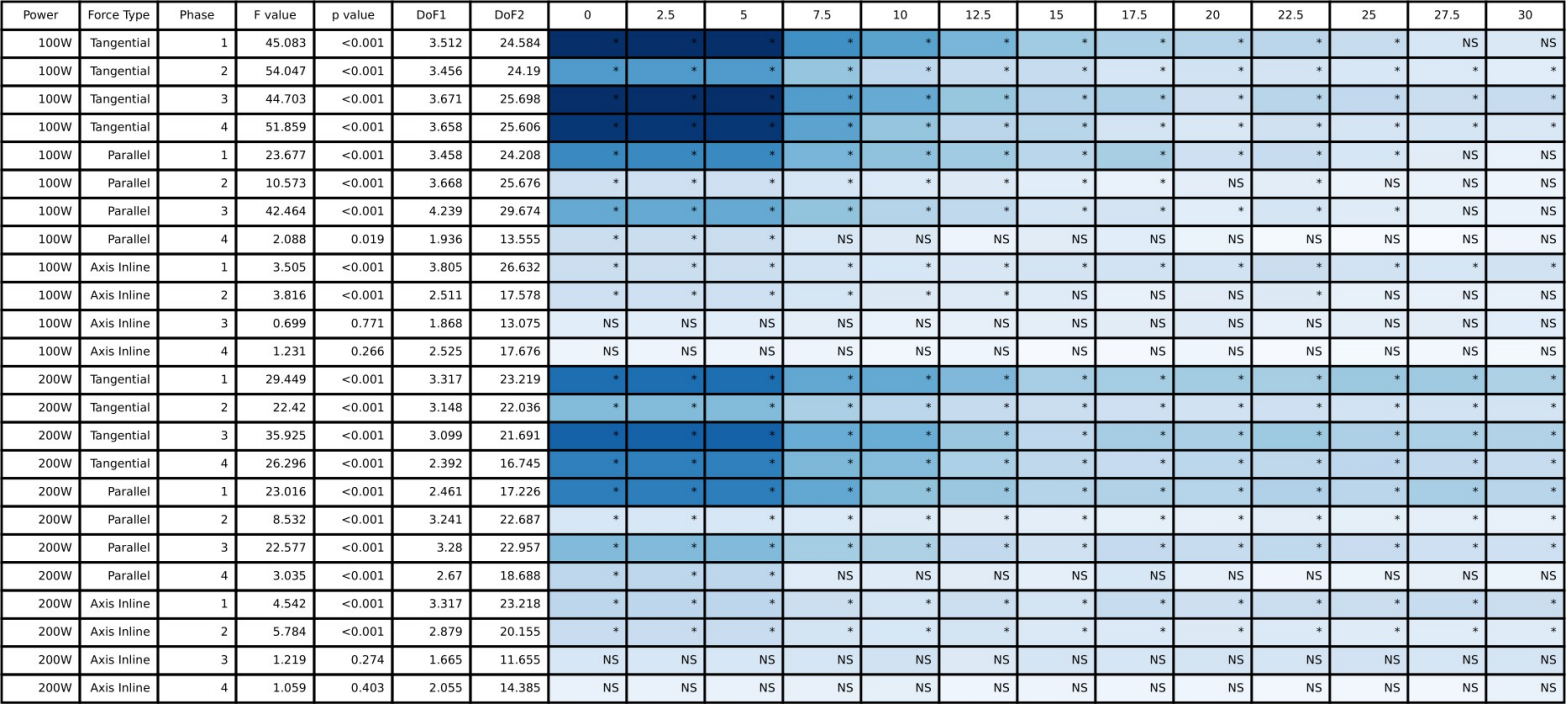
The ANOVA results for the mean pedal force data and the post hoc Dunnett’s analysis for the different counterweight conditions relative to double-leg cycling. The blue shading represents the effect size with darker blue representing largest effect size for this table and white would indicate an effect size of 0. NS indicates a non-significant post hoc test and an asterisk indicates a significant post hoc test.

**Fig 7:**
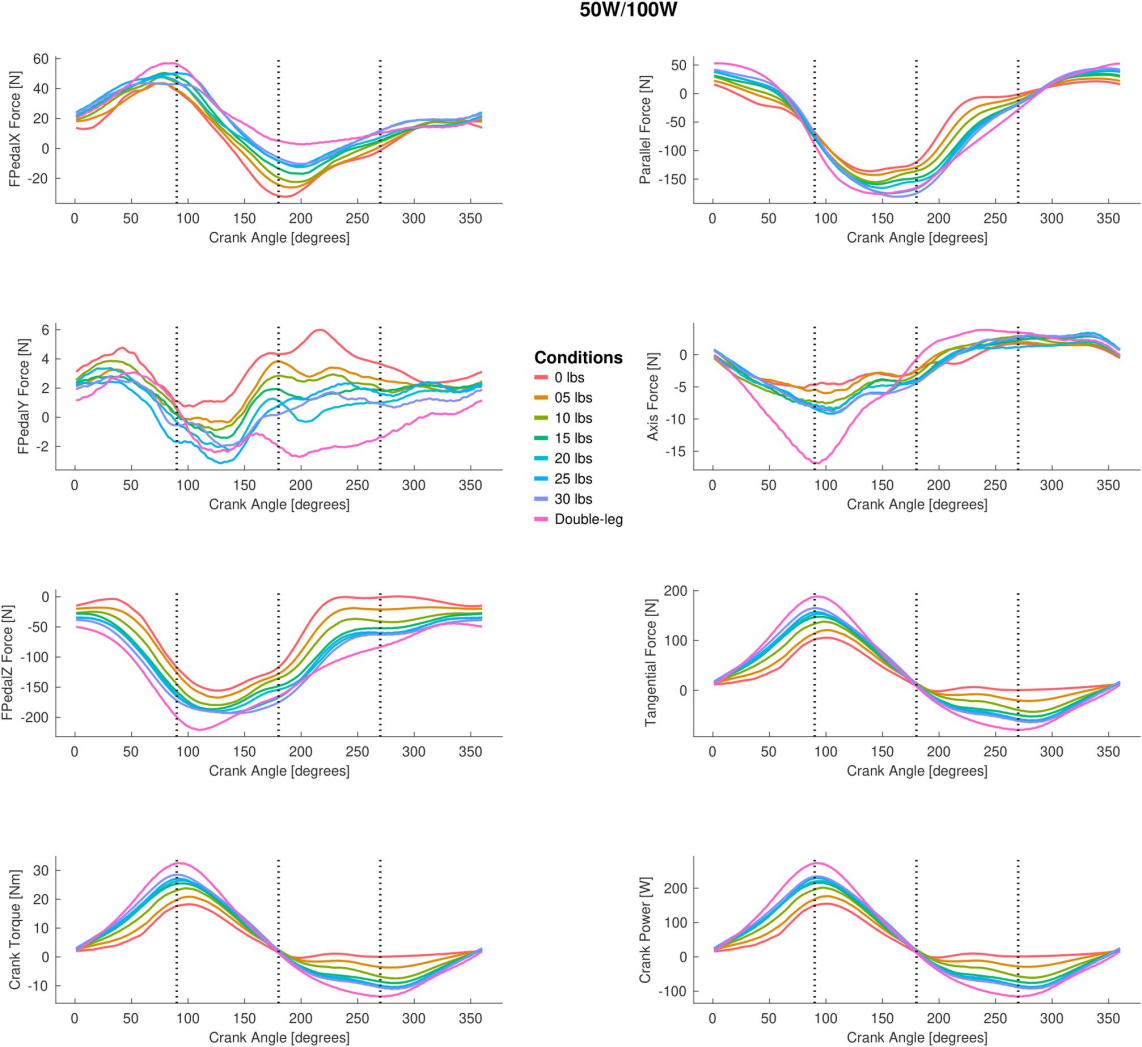
The group mean pedal data for different single-leg cycling conditions and double leg cycling across a pedal revolution for 50W single leg cycling (100W double leg cycling). The left column displays the pedal data in the pedal reference frame. X represents the anterior (+) and posterior (-) force, Y represents the medial (+) and lateral (-) force, and Z represents the vertical superior (+) and inferior (-) force. The bottom left figure shows the crank torque. The right column represents the pedal forces relative to the crank. Parallel represents the anterior (+) and posterior (-) force, tangential represents the clockwise (+) and counterclockwise (-) force, and axis represents the medial (+) and lateral (-) force. Data are not displayed for all single-leg cycling counterweight conditions for sake of display clarity.

**Fig 8:**
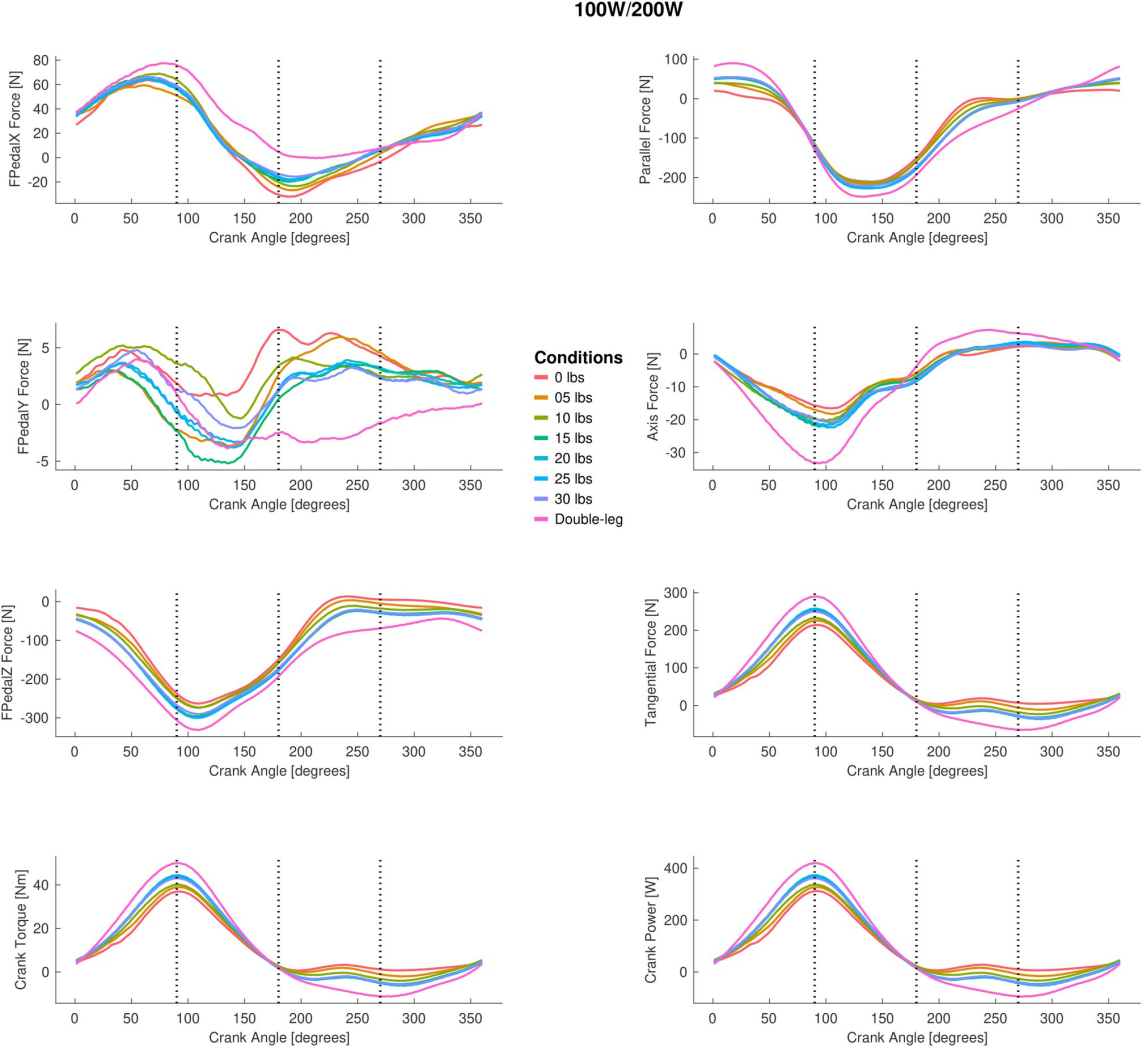
The group mean pedal data for different single-leg cycling conditions and double leg cycling across a pedal revolution for 100W single leg cycling (200W double leg cycling). The left column displays the pedal data in the pedal reference frame. X represents the anterior (+) and posterior (-) force, Y represents the medial (+) and lateral (-) force, and Z represents the vertical superior (+) and inferior (-) force. The bottom left figure shows the crank torque. The right column represents the pedal forces relative to the crank. Parallel represents the anterior (+) and posterior (-) force, tangential represents the clockwise (+) and counterclockwise (-) force, and axis represents the medial (+) and lateral (-) force. Data are not displayed for all single-leg cycling counterweight conditions for sake of display clarity.

The full tangential, parallel, and in line with the crank axis pedal force ANOVA results are presented in Fig 5 (peak-to-peak) and Fig 6 (mean), and the group mean pedal tangential, parallel, and in line with crank axis forces throughout the pedal stroke are plotted in Fig 7 (50/100 W) and Fig 8 (100/200 W). Peak-to-peak and mean tangential pedal forces were significantly different for both power outputs in all phases. Peak-to-peak and mean parallel pedal forces were significantly different across all phases at 50/100 W and for the 100/200 W condition, these same variables were different across all phases except for peak-to-peak parallel pedal forces in Phases 2 and 3 at 100/200 W. Peak-to-peak pedal forces in line with the crank axis were significantly different for all phases at 50/100W and Phases 1, 2, and 4 at 100/200 W, but mean pedal forces in line with the crank axis were only significantly different in Phases 1 and 2 for both 50/100 W and 100/200 W. See Figs 5 and 6 for post hoc test results for these significant main effects.

### 3.3 Joint moments

**Fig 9:**
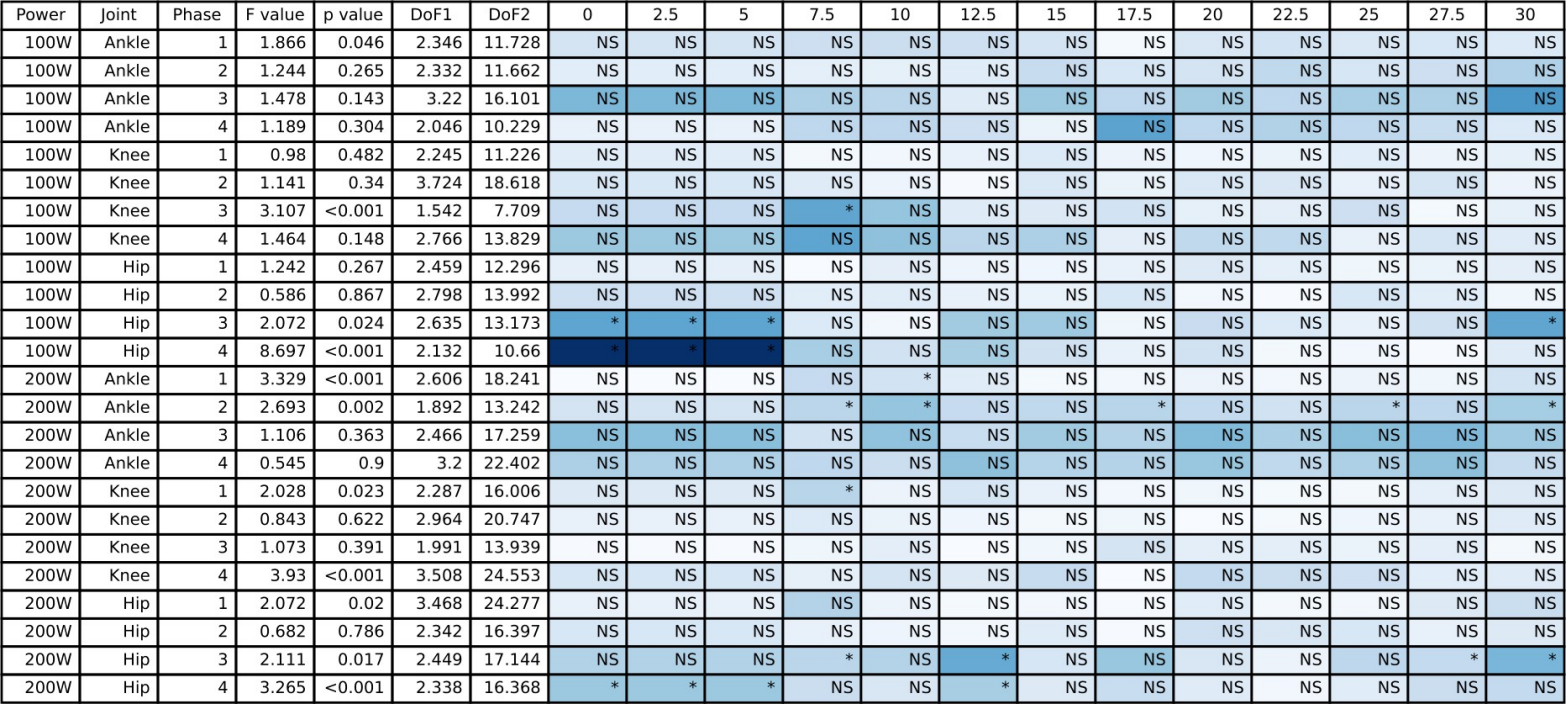
The ANOVA results for the peak-to-peak joint moment data and the post hoc Dunnett’s analysis for the different counterweight conditions relative to single-leg cycling. The blue shading represents the effect size with darker blue representing largest effect size for this table and white would indicate an effect size of 0. NS indicates a non-significant post hoc test and an asterisk indicates a significant post hoc test.

**Fig 10:**
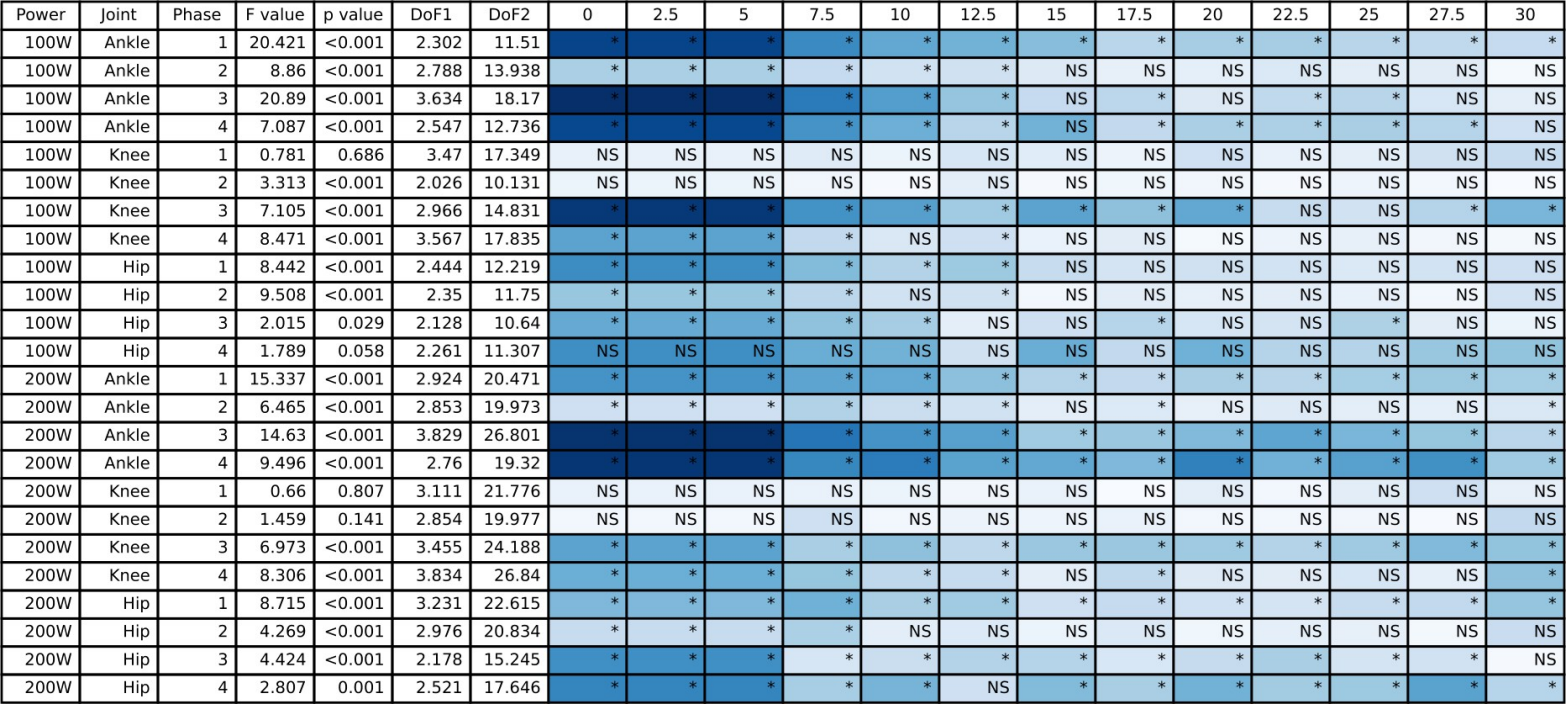
The ANOVA results for the mean joint moment data and the post hoc Dunnett’s analysis for the different counterweight conditions relative to single-leg cycling. The blue shading represents the effect size with darker blue representing largest effect size for this table and white would indicate an effect size of 0. NS indicates a non-significant post hoc test and an asterisk indicates a significant post hoc test.

The full joint moment ANOVA results for the hip, knee, and ankle are presented in Fig 9 (peak-to-peak) and Fig 10 (mean), and the group mean joint moments for the hip, knee, and ankle throughout the pedal stroke are plotted in Fig 3 (50/100 W) and Fig 4 (100/200 W). Peak-to-peak joint moments were significantly different at the ankle at 50/100 W in Phase 1 only and at 100/200 W in Phases 1 and 2 only. In contrast, mean ankle joint moments were significantly different for all Phases at 50/100 W and 100/200 W. Peak-to-peak knee joint moments were only significantly different in Phase 3 at 50/100 W and in Phases 1 and 4 at 100/200 W, but mean knee joint moments were significantly different in Phases 2–4 at 50/100 W and Phases 3 and 4 at 100/200 W. At the hip, peak-to-peak joint moments were significantly different in Phases 3 and 4 at 50/100 W and Phases 1, 3, and 4 at 100/200 W. In contrast, mean hip joint moments were significantly different for all Phases and power outputs except for Phase 4 at 50/100 W. See Figs 9 and 10 for post hoc test results for these significant main effects.

### 3.4 Joint work

**Fig 11:**
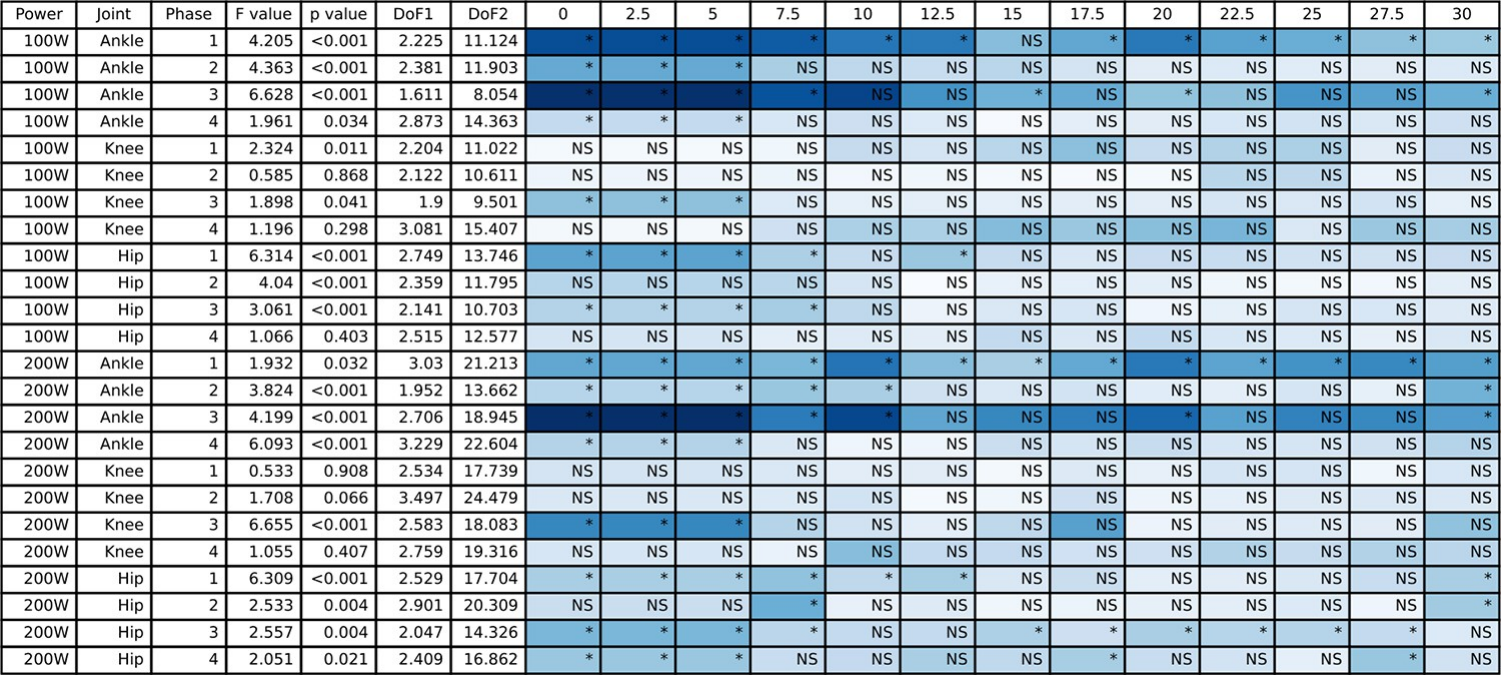
The ANOVA results for the positive work data and the post hoc Dunnett’s analysis for the different counterweight conditions relative to single-leg cycling. The blue shading represents the effect size with darker blue representing largest effect size for this table and white would indicate an effect size of 0. NS indicates a non-significant post hoc test and an asterisk indicates a significant post hoc test.

**Fig 12:**
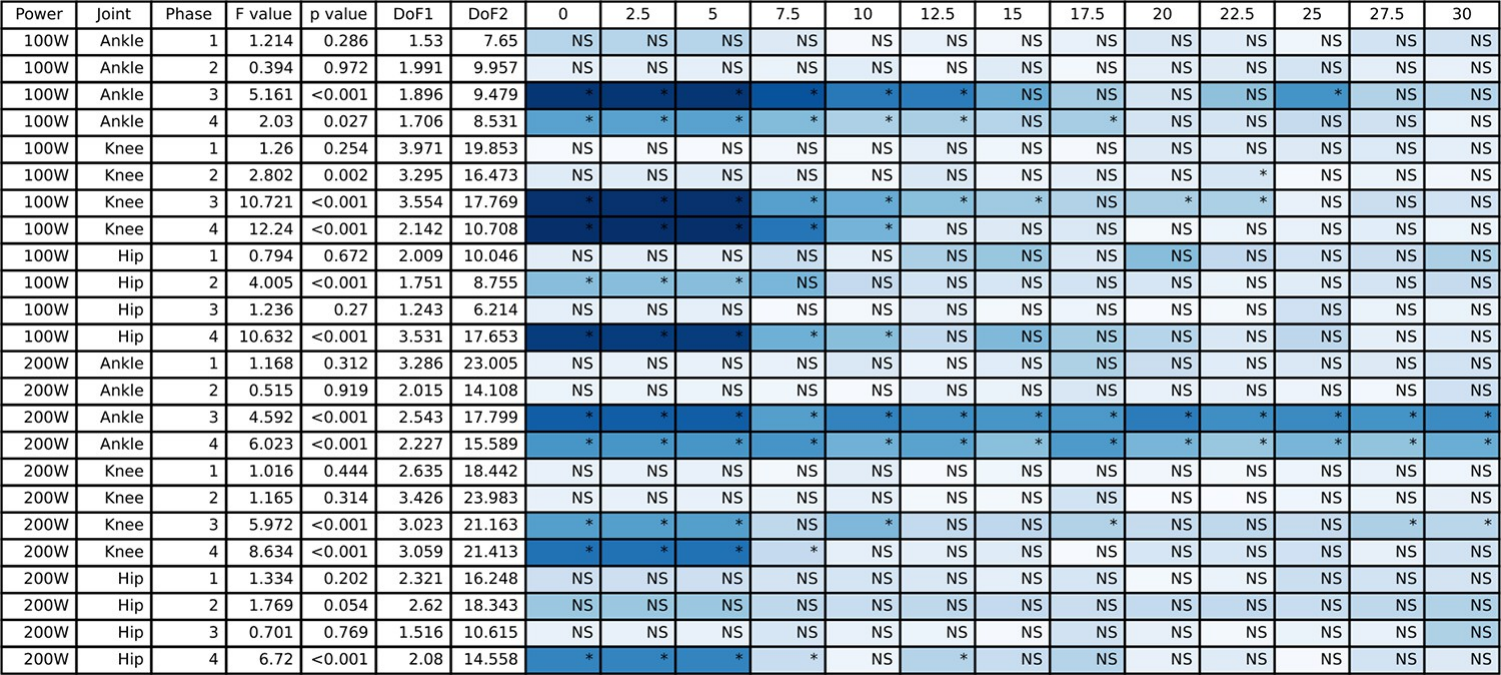
The ANOVA results for the negative work data and the post hoc Dunnett’s analysis for the different counterweight conditions relative to single-leg cycling. The blue shading represents the effect size with darker blue representing largest effect size for this table and white would indicate an effect size of 0. NS indicates a non-significant post hoc test and an asterisk indicates a significant post hoc test.

All joint work ANOVA results for the hip, knee, and ankle are presented in Fig 11 (positive work) and Fig 12 (negative work), and the group mean joint power for the hip, knee, and ankle throughout the pedal stroke are plotted in Fig 3 (50/100 W) and Fig 4 (100/200 W). Positive work at the ankle was significantly different for all four Phases at both 50/100 W and 100/200 W, whereas negative work at the ankle was only significantly different during Phases 3 and 4 at both 50/100 W and 100/200 W. At the knee, positive work was significantly different during Phases 1 and 3 at 50/100 W and Phase 3 at 100/200 W, but negative work at the knee was significantly different during Phases 2–4 at 50/100 W and Phases 3 and 4 at 100/200 W. Positive work at the hip was significantly different during Phases 1–3 at 50/100 W and all Phases at 100/200 W, with negative work being significantly different during Phases 2 and 4 at both 50/100 W and 100/200 W. See Figs 11 and 12 for post hoc test results for these significant main effects.

**Fig 13:**
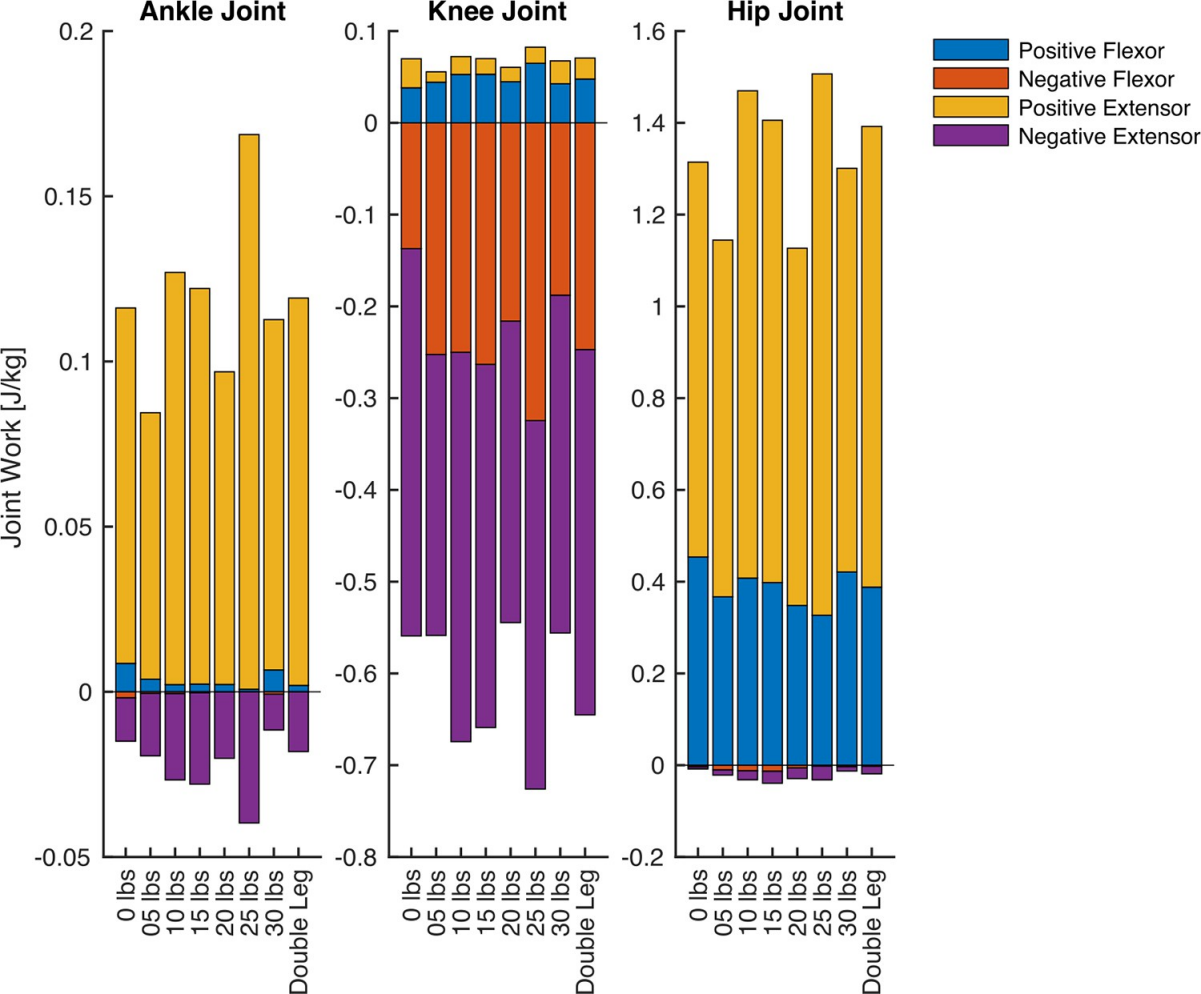
The group mean data for the joint work during 50W single leg cycling (100W double leg cycling) separated into flexor positive work (blue), flexor negative work (red), extensor positive work (yellow), and extensor negative work (purple). The left figure shows the ankle, the middle figure shows the knee, and the right figure shows the hip data. Data are not displayed for all single-leg cycling counterweight conditions for sake of display clarity.

**Fig 14:**
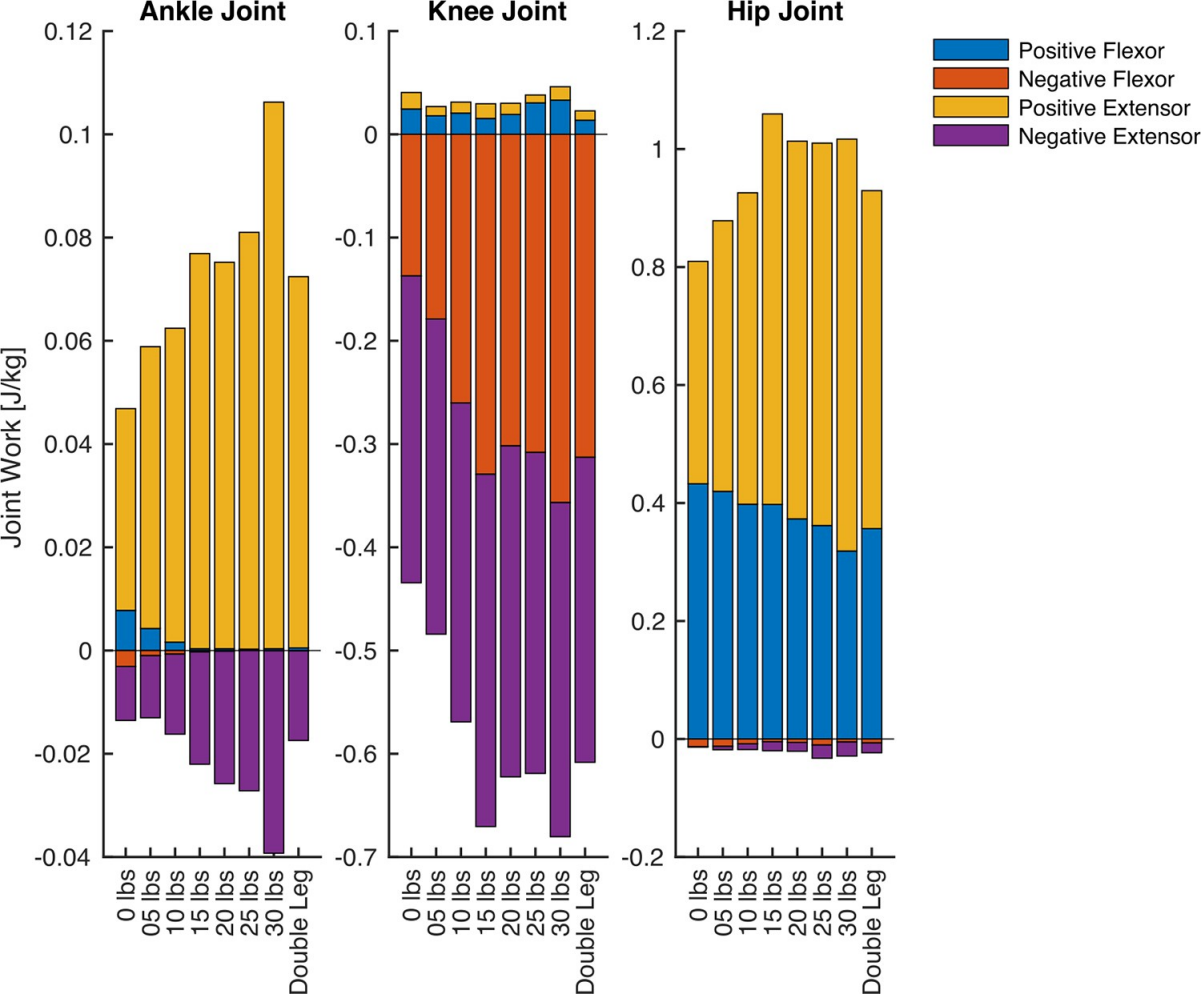
The group mean data for the joint work during 100W single leg cycling (200W double leg cycling) separated into flexor positive work (blue), flexor negative work (red), extensor positive work (yellow), and extensor negative work (purple). The left figure shows the ankle, the middle figure shows the knee, and the right figure shows the hip data. Data are not displayed for all single-leg cycling counterweight conditions for sake of display clarity.

In addition to the reporting of the joint negative and power work, we descriptively displayed the further discretization of negative and positive work into the flexor positive and negative joint work and extensor positive and negative work for the three joints analyzed (50/100W: Fig 13; 100/200W: Fig 14). We also present the data for the 2-way ANOVA in which the single-leg cycling data was normalized by the double-leg cycling data for each variable of interest in the supporting information (S1 File). For this supplement, the 2-way ANOVA included the power outputs (0-30lbs) and the different power outputs (50W and 100W). Each dependent variable for the 2-way ANOVA was represented as a ratio of its value in the double-leg cycling condition.

## 4.0 Discussion

The present study investigated the effect of counterweight mass on the 3D kinematics and kinetics (hip, knee, ankle) of single-leg cycling in comparison to double-leg cycling at the same relative power output (i.e., W per active leg). The main findings are that peak-to-peak joint angles were largely similar for single-leg and double-leg cycling and that relatively heavy counterweights were needed, but not always sufficient, to attenuate differences in pedal forces, joint moments, and joint work between double-leg cycling and single-leg cycling. These findings agree with previous studies that investigated the effect of one counterweight mass [18–20] on 2D kinetics and kinematics, but we used 3D motion capture and 3D instrumented pedals along with model-constrained optimization to provide a more in-depth biomechanics analysis. Our results extend previous findings by providing empirical support for the use of relatively heavy counterweights for single-leg cycling. That the heavier counterweights were insufficient to match double-leg cycling suggests that the two cycling modes are distinct in subtle ways that may not be equalized with a static counterweight on the contralateral pedal; however, the implications of this suggestion depend on the application.

The number of counterweight masses investigated in the present study makes it unique relative to previous studies [18–20] that compared double-leg cycling and unassisted single-leg cycling to one condition of counterweighted single-leg cycling (10–11.64 kg; ~22–25 lbs). We acknowledge that the unit kg for mass should be used, but for the context of this discussion and to compare across studies, we will use the imperial unit of lb. A common finding among studies was that the addition of a counterweight reduces, but does not fully eliminate, dissimilarities between double-leg and single-leg cycling with respect to kinetics and kinematics [18–20]. The counterweight accomplishes this feat by storing energy during the pedal downstroke marked by leg extension and releasing energy during the pedal upstroke marked be leg flexion. Intuitively, one might expect that additional force would need to be applied during the perceived “push”/downstroke phases (Phases 1 and 2) to “lift” the counterweight on the contralateral pedal; however, our data indicated that there are lower tangential crank forces for all single-leg cycling conditions during the “push”/downstroke phases, indicating that more force was produced for double-leg cycling in these phases. A simple explanation for this result is that the “inactive” leg in a double-leg cycling task applies an opposing force (i.e., counterclockwise) during the “recovery”/upstroke phases (Phases 3 and 4), and this negative work on the contralateral crank needs to be countered by the “active” leg. In unassisted single-leg cycling, there is less tangential crank force in the “push” phases, likely because there is less negative work (or no internal work performed by the contralateral limb acting on the crank) being performed by the vacant contralateral pedal. Instead, active ipsilateral leg flexion is needed during the “recovery” phases of unassisted single-leg cycling (i.e., 0lb condition) [18–20]. Increasingly heavy counterweights increased the positive tangential crank forces in the “push” phases, presumably by simultaneously increasing the force opposite to the intended crank direction on the contralateral pedal; however, even the heaviest, 30-lb condition was insufficient to match double-leg cycling, suggesting the simple addition of a static counterweight did not perform as much negative work–or at least not the same pattern of negative power–as the inactive leg during double-leg cycling.

In agreement with our results, Elmer et al. [18] also reported that a counterweight (11.64 kg; ~25 lbs) reduced active knee and hip flexion and ankle dorsiflexion and increased hip extension and ankle plantarflexion work during single-leg cycling, resulting in significantly lower overall leg flexion work for counterweight single-leg cycling (i.e., having a higher counterweight mass of 25lbs) versus unassisted single-leg cycling (i.e., equivalent to our 0 lbs condition). Similar to our study, the “counterweight” mass used by Elmer et al. did not fully replicate double-leg cycling and also showed less leg flexion positive work by the hip and the ankle, particularly for the 100/200W condition (see Fig 13). Previous work examining kinematics and kinetics of single-leg cycling have also shown similar findings of joint powers and joint work during single-leg cycling never matching double-leg cycling [18, 19]. These studies used a 2D analysis, and our study incorporated a more comprehensive 3D analysis. Although the same conclusion is drawn that single-leg cycling never matches double-leg cycling, the magnitudes and direction of joint power were different in our study. Potentially because of these study design changes, we did observe differences in direction and magnitudes of time-series joint power data relative to these other studies, particularly at the knee. These differences could have emerged due to the 3D analysis or the inclusion of internal joint moments and power in our study–whether the internal joint moment was used in previous studies was unclear. Our knee joint power data resembles that of other cycling studies using a 3D analysis and reporting internal joint moments and powers [25]. Nevertheless, our study shows the same general findings as previous work in the field. From our incremental approach, it seems that even heavier (impractically so) counterweight masses may be needed to achieve similar cycling biomechanics.

We hypothesized that we would find an optimal counterweight condition that resulted in single-leg cycling biomechanical measures that were equivalent to double-leg cycling. Essentially, we expected to identify a “goldilocks” condition that would match double-leg cycling and that counterweights too light or too heavy would cause deviations from optimal double-leg cycling biomechanics. The results of this study did not support our main hypothesis, and one of the main findings is that the biomechanical measures in even the heaviest counterweight condition (i.e., 30 lbs) did not emulate double-leg cycling but oftentimes produced measures that were the most similar to double-leg cycling. The reason for this finding is unknown, but we identified several possible explanations. The first reason is the mass of our participants’ “inactive” legs. Using the participant-specific scaled *OpenSim* models, we estimated the mass of each participant’s leg (see S1 Table). On average, the mass of the lower limb (thigh, shank, foot) was 14.3 kg (SD: 1.7), which equates to 31.6 lbs (SD: 3.7). This approximate mass of the lower limb is closest to the largest counterweight condition, and we can attribute part of the similarity between 30 lbs single-leg cycling to double leg cycling to this effect. We speculate that if a higher counterweight was tested, an “optimal” counterweight that was closer to double leg cycling may have been evident; however, adding more mass to the counterweight would be difficult and possibly infeasible with the crank set-up in this study. To test this hypothesis without collecting additional experimental data (as we could not with our crank and pedal set-up), we performed an additional analysis. Specifically, we performed an exploratory linear regression not included in our initial experimental design on the peak force tangential to the crank data for the single-leg cycling conditions and solving for the same peak tangential force measure for double leg cycling. From the exploratory analysis, it was predicted that the counterweight condition would have to be ~50 lbs (~22.7 kg) to match the double leg cycling data. Even with this prediction, the pedal forces may never match one another. The reason we believe that this high counterweight (~50 lbs) may not be sufficient is that the manner or pattern that the counterweight contributes to crank moment/torque is another, and our second, plausible reason that single leg-cycling data never attained near identical patterns of double-leg cycling. We modelled the torque that each counterweight would provide to the crank given the mass, crank length, and crank angle (see S2 Fig). Descriptively, the torque/moment of the counterweight is much different than the crank torque/moment applied by the participant during double-leg cycling (see Figs 4 and 5). One hypothetical method to improve the torque/moment applied to the crank by the counterweight would be to implement a robotic device that applies a torque/moment to the other crank during single-leg cycling or an adaptive crank that changes length during the upstroke to reduce the overall crank torque/moment during the upstroke (Phase 3 and 4) and increase it during the downstroke (Phases 1 and 2) (see S2 Fig). We suggest that these two reasons explain dissimilarities between double- and single-leg cycling.

The results did not converge on one universal “counterweight” mass for all applications of single-leg cycling. By examining the pedal data (see Figs 5 and 6), there were minimal variables that were not significantly different than double-leg cycling and may cause one to conclude that single-leg cycling is never suitable for mimicking double-leg cycling. These pedal force measures are meaningful, but a better representation of the amount of muscle force produced during single-leg cycling is to examine the joint moment and joint work data, given the peak-to-peak joint angle data remained largely unchanged. Examining these data, it is evident that any counterweight condition between 0 and 5 lbs is not suitable for single-leg cycling. Above 5 lbs, we start to see convergence of a number of joint moment and joint work variables with double-leg cycling (50/100W: Phase 2 positive ankle work, Phase 3 positive knee work; 200W: Phase 2 hip moment). For the 15 lbs condition, we see trends of the single-leg cycling biomechanical variables (e.g., at 50/100W: Phase 2 ankle and hip moment and Phase 4 knee moment) being not different from double-leg cycling, and this lack of difference remains up to the heaviest, 30 lbs conditions. From this perspective, a liberal estimate would be to use a counterweight of at least 15 lbs or higher. To be more conservative using the joint moment and work data, a counterweight of at least 20 lbs is recommended, which agrees with the majority of published studies [6, 10, 18–20, 26]. This study provides new, additional information such that a reference table is provided, allowing researchers to see what counterweight condition should be used for their applications and intended exercise stimulus.

The extent to which the biomechanical dissimilarities of single-leg and double-leg cycling are meaningful likely depends on the application. In exercise physiology studies, single-leg cycling is often used as a means to reduce the active muscle mass for a given task to study one or more physiological systems under different levels of stress from the same general mode of exercise [27–29]. In this context, the biomechanical differences demonstrated between cycling modes in this study may be important if they directly impact the variables of interest. Other exercise physiology studies use single-leg cycling as a method to train the legs of individuals independently, for example, to compare the effects of different exercise stimuli on skeletal muscle adaptations to exercise [2, 26], or to train one leg while the other remains untrained [30, 31] and examine the effects of training on physiological responses to exercise. In these cases, biomechanical differences would not likely impact the internal validity of the results, but they may affect the transferability of those results to double-leg cycling. Biomechanical differences between cycling modes may be important in the context of athletic training, as the principle of training specificity indicates that a training stimulus will be more effective for improving performance if it closely matches the performance task. Given that a greater relative power output can be performed by cyclists during single-leg cycling, which provides a means to increase oxidative capacity of skeletal muscle [10], the limitations in transferability to a different task may be outweighed by the positive effects of the adaptations incurred. Lastly, the use of single-leg cycling in patient populations [4] is probably not diminished by the biomechanical differences between single-leg and double-leg cycling. For these applications, single-leg cycling is employed because it is more tolerable than double-leg cycling (e.g., for patients with COPD [11]) or because one leg is unable to train (e.g., for patients recovering from ACL surgery [13]). For all situations, a relatively heavier counterweight is still likely beneficial, as heavier counterweights require more positive extension work relative to lighter counterweights (Figs 13 and 14, ankle and hip) and counterweights induce less cardiorespiratory stress than unassisted single-leg cycling [5], potentially making it a better training stimulus for the quadriceps and gluteal muscles than unassisted single-leg cycling. Alternatively, a fixed-gear cycle ergometer [11] or simply using one’s own “inactive” leg as the counterweight [7] may also be effective. Overall, minimizing biomechanical differences between cycling modes is advantageous, but most applications of single-leg cycling are probably not diminished by the biomechanical differences that are seemingly unavoidable, even for heavier counterweights.

As with any study, the inferences drawn from this study come with limitations. The first limitation is the period of learning the new task. Single-leg cycling is a novel task that individuals may not have performed previously. For this reason, we had participants visit the lab for a familiarization session. This session is typical of what would be performed in a single-leg cycling intervention as seen elsewhere [32]. With that being said, full learning of a task does not occur only within a single session. There could be additional longer-term changes that could occur over repeated sessions. To mitigate any effects that learning had on the participants’ biomechanics, we randomized the counterweight conditions such that one counterweight condition was not overrepresented at an earlier block with less adaptation or at a later block which would have more adaptation. The cadence (crank angular velocity) implemented in this study, which matched previous single-leg cycling studies [5, 18], is another main limitation that may affect the generalizability of our results. It could be that with lower or higher cadences (i.e., pedaling frequency) the effects of counterweight on a person’s cycling biomechanics may differ and the resulting optimal counterweight to mimic double-leg cycling could be different than our conclusions. Also, we did examine two different power outputs for single-leg cycling (S1 File), but did not notice any large differences in our reported results between these power outputs. It could be with larger power outputs, there may be more important interaction effects of counter-weight mass and cycling power output. Future research should examine similar research questions, except have participants cycle at different pedaling frequencies, a higher power output or an external cycling load that is normalized to the person’s body mass. Lastly, although single-leg cycling interventions can be applied to individuals without any clinical conditions, the participants in this study and the inferences made from this study may not represent the biomechanical changes that may occur with a clinical population that would benefit from a single-leg cycling intervention.

## 5.0 Conclusion

Single-leg cycling with a static counterweight from 0 to 30 lbs does not perfectly match double leg cycling biomechanics. If the goal of applying single-leg cycling is to perfectly emulate double-leg cycling, a static counterweight up to 30 lbs seems to be inadequate; however, single-leg cycling biomechanics begin to converge on double-leg cycling with the use of relatively heavy counterweights. If the purpose is to approximate the biomechanics of double-leg cycling with single-leg cycling (e.g., for an exercise training intervention), it appears that using a counterweight > 15lbs is sufficient but >20lbs might be better to ensure the biomechanics of single-leg cycling are similar to that of double-leg cycling. Thus, the typical range of counterweight masses in the literature appears to be supported by our empirical data and we have added a reference table to choose a counterweight that is suitable for researchers’ applications. Future work should examine single-leg cycling at different cadences and aim to develop new technologies to perfectly match single-leg cycling to double-leg cycling biomechanics.

## Supporting information

S1 Fig Descriptive statistics. The ANOVA results for the pedal data, joint angles, joint moments, and joint powers (Figs 2, 5, 6, 9–12). Each cell for the counterweight mass shows the mean, standard deviation, and effect size from Hedge’s *g*. The title describes the dependent variable that was input into the ANOVA.
(PDF)

S2 Fig Modelled crank effects. The static crank data (top figure) models the effects of the different counterweights used in this study on crank torque depending on the different crank angle and given a constant crank length. The adaptive crank data models the effects of the different counterweights used in this study on crank torque depending on the different crank angle and given a changing crank length that increases by an order of 2 on the downstroke and decrease by an order of 2 during the upstroke.
(TIFF)

S1 Table Estimated leg mass. The table represents the estimated mass of the leg for each participant as well as the group mean and standard deviation.
(TIFF)

S1 File 2-way ANOVA. The 2-way ANOVA for counterweight mass and power output for single-leg cycling (i.e., 50W, 100W). The single-leg cycling data which was normalized by the double-leg cycling data for each variable of interest.
(PDF)
