# Supplementary material for: Counterweight mass influences single-leg cycling biomechanics
Source: PLoS One. 2024 Jun 7;19(6):e0304136. doi: 10.1371/journal.pone.0304136 (PMC11161077; doi:10.1371/journal.pone.0304136)
Supplement: S1 Fig — The ANOVA results for the pedal data, joint angles, joint moments, and joint powers (Figs 2, 5, 6, 9–12). Each cell for the counterweight mass shows the mean, standard deviation, and effect size from Hedge’s g. The title describes the dependent variable that was input into the ANOVA. (PDF) [file pone.0304136.s001.pdf]

Peak to Peak Joint Angle ANOVA

Power	Joint	Phase	F value	p value	DoF1	DoF2	0lbs	2.5 lbs	5 lbs	7.5 lbs	10 lbs	12.5 lbs	15 lbs	17.5 lbs	20 lbs	22.5 lbs	25 lbs	27.5 lbs	30 lbs
100W	Ankle	1	5.056	<0.001	2.727	13.635	10.5(4.4);0.3	10.5(4.4);0.3	10.5(4.4);0.3	8.0(2.6);0.2	7.5(3.2);0.3	7.7(4.0);0.3	9.1(4.4);0.1	6.1(4.2);0.5	6.4(2.5);0.6	6.6(3.2);0.5	7.2(3.5);0.4	6.3(2.7);0.6	6.8(3.5);0.4
100W	Ankle	2	4.397	<0.001	2.939	14.693	8.3(3.4);1.2	8.3(3.4);1.2	8.3(3.4);1.2	10.7(5.2);0.4	11.1(4.7);0.4	11.7(5.2);0.1	10.1(3.5);0.9	11.1(4.1);0.4	12.2(5.0);0.1	11.9(5.3);0.2	10.9(5.3);0.3	12.1(4.1);0.2	11.4(4.7);0.3
100W	Ankle	3	1.318	0.219	2.564	12.819	7.5(3.0);0.3	7.5(3.0);0.3	7.5(3.0);0.3	7.8(3.6);0.2	8.0(4.5);0.2	7.3(4.4);0.4	8.4(3.4);0.4	7.7(4.7);0.3	8.5(5.0);0.0	7.0(4.2);0.4	7.0(4.2);0.3	7.8(4.9);0.2	8.4(5.3);0.1
100W	Ankle	4	1.224	0.278	3.039	15.195	9.1(3.9);0.1	9.1(3.9);0.1	9.1(3.9);0.1	9.2(4.5);0.1	9.2(4.4);0.1	8.6(3.3);0.1	8.6(4.0);0.1	6.8(2.2);0.5	9.4(3.9);0.1	9.5(4.1);0.2	8.3(3.1);0.2	8.7(4.2);0.0	9.3(3.6);0.1
100W	Knee	1	2.37	0.009	2.257	11.284	48.5(8.6);0.3	48.5(8.6);0.3	48.5(8.6);0.3	48.1(7.2);0.2	47.9(6.3);0.2	44.4(8.2);0.3	45.2(8.5);0.2	49.8(5.5);0.2	44.9(5.5);0.2	44.3(9.6);0.2	46.7(6.9);0.0	45.8(6.3);0.1	45.4(5.7);0.1
100W	Knee	2	1.171	0.316	2.759	13.795	26.0(6.5);0.1	26.0(6.5);0.1	26.0(6.5);0.1	25.2(5.8);0.2	25.5(5.2);0.2	31.1(11.0);0.4	28.2(8.3);0.1	24.6(5.2);0.0	28.8(7.6);0.3	29.0(9.0);0.3	26.1(6.9);0.1	27.4(4.3);0.1	27.8(4.8);0.2
100W	Knee	3	0.871	0.592	1.475	7.375	51.3(8.3);0.3	51.3(8.3);0.3	51.3(8.3);0.3	52.2(7.1);0.4	52.2(8.0);0.4	47.1(12.5);0.2	49.9(10.2);0.1	54.3(6.0);0.5	50.4(6.8);0.2	49.5(11.9);0.1	51.4(8.3);0.3	51.4(7.8);0.3	51.2(6.6);0.3
100W	Knee	4	2.741	0.003	2.153	10.767	16.9(8.6);0.5	16.9(8.6);0.5	16.9(8.6);0.5	18.6(6.0);0.4	19.3(5.8);0.3	25.1(11.9);0.3	22.0(8.6);0.0	18.0(3.9);0.2	22.7(7.0);0.2	22.7(10.1);0.1	19.4(6.7);0.2	21.0(4.7);0.1	22.3(5.7);0.1
100W	Hip	1	0.96	0.502	2.599	12.997	25.8(5.8);0.2	25.8(5.8);0.2	25.8(5.8);0.2	25.4(8.4);0.1	24.8(6.1);0.0	23.9(9.0);0.2	24.4(5.2);0.2	26.9(6.1);0.0	23.0(3.8);0.4	22.2(6.0);0.4	25.3(7.4);0.0	22.8(6.6);0.3	23.5(6.1);0.2
100W	Hip	2	1.433	0.161	2.53	12.649	19.9(6.2);0.1	19.9(6.2);0.1	19.9(6.2);0.1	20.0(4.3);0.1	20.7(5.5);0.0	21.7(4.6);0.2	22.6(8.6);0.3	21.1(5.4);0.1	23.2(7.6);0.4	23.0(6.8);0.4	20.8(4.8);0.1	23.2(5.1);0.5	22.2(6.2);0.3
100W	Hip	3	1.702	0.075	2.972	14.858	22.4(6.4);0.1	22.4(6.4);0.1	22.4(6.4);0.1	22.0(7.9);0.1	21.6(7.3);0.0	21.3(8.8);0.1	22.0(5.7);0.1	22.0(8.0);0.0	19.3(5.7);0.3	19.9(6.0);0.2	21.3(7.3);0.1	20.5(7.3);0.1	21.1(8.5);0.1
100W	Hip	4	5.73	<0.001	2.548	12.741	20.4(4.7);0.7	20.4(4.7);0.7	20.4(4.7);0.7	22.2(4.3);0.4	23.0(4.9);0.2	23.8(4.0);0.1	23.4(6.8);0.1	24.8(4.7);0.1	25.1(6.0);0.1	25.7(5.3);0.2	23.7(4.0);0.0	25.1(4.2);0.2	23.5(6.8);0.1
200W	Ankle	1	1.914	0.034	3.302	23.117	7.7(2.6);0.1	7.7(2.6);0.1	7.7(2.6);0.1	7.6(3.5);0.1	6.3(2.5);0.3	7.4(4.0);0.1	6.7(3.5);0.1	6.2(3.6);0.1	6.0(2.9);0.3	6.1(3.0);0.3	6.2(3.1);0.3	5.3(1.5);0.5	6.2(3.0);0.1
200W	Ankle	2	2.752	0.002	3.797	26.576	11.2(4.5);0.4	11.2(4.5);0.4	11.2(4.5);0.4	11.4(5.3);0.4	11.3(3.8);0.4	12.8(5.1);0.1	11.8(3.4);0.3	13.4(5.2);0.1	12.8(4.8);0.1	13.1(4.2);0.0	12.2(4.4);0.2	14.2(5.2);0.2	10.7(4.8);0.6
200W	Ankle	3	1.066	0.398	3.733	26.134	8.0(3.0);0.1	8.0(3.0);0.1	8.0(3.0);0.1	9.4(5.4);0.2	6.4(2.8);0.6	8.0(3.4);0.1	6.2(2.1);0.7	6.6(3.4);0.5	7.2(4.1);0.3	7.5(2.8);0.3	7.7(3.9);0.2	7.3(4.8);0.2	6.6(3.1);0.5
200W	Ankle	4	0.525	0.913	3.606	25.241	9.7(6.3);0.1	9.7(6.3);0.1	9.7(6.3);0.1	8.1(2.7);0.5	9.6(5.1);0.1	8.9(4.2);0.3	9.0(4.1);0.2	8.6(4.0);0.1	9.7(3.8);0.1	9.9(4.3);0.0	9.5(4.7);0.1	9.7(3.9);0.1	10.1(4.9);0.0
200W	Knee	1	1.331	0.203	3.86	27.021	49.4(6.0);0.4	49.4(6.0);0.4	49.4(6.0);0.4	47.9(9.8);0.2	45.9(8.6);0.1	46.1(7.7);0.0	49.2(6.2);0.3	47.6(8.5);0.2	49.1(4.7);0.4	49.0(6.4);0.3	49.8(5.3);0.4	48.5(5.9);0.2	48.2(8.0);0.0
200W	Knee	2	1.867	0.039	3.321	23.249	26.4(5.1);0.2	26.4(5.1);0.2	26.4(5.1);0.2	27.1(9.0);0.2	28.3(7.1);0.4	31.6(9.9);0.7	25.4(6.3);0.1	28.9(6.8);0.9	26.1(4.3);0.1	25.4(6.0);0.0	24.4(5.5);0.1	27.1(5.9);0.3	25.2(6.6);0.3
200W	Knee	3	1.11	0.359	1.497	10.481	52.6(7.4);0.4	52.6(7.4);0.4	52.6(7.4);0.4	49.0(12.0);0.1	49.8(11.9);0.2	48.1(10.7);0.0	52.9(7.5);0.4	51.9(10.9);0.0	53.6(6.7);0.6	52.2(8.0);0.4	53.4(6.1);0.5	53.4(7.1);0.5	50.5(8.3);0.1
200W	Knee	4	1.947	0.03	2.918	20.427	18.1(4.6);0.3	18.1(4.6);0.3	18.1(4.6);0.3	22.6(12.0);0.2	22.8(10.6);0.2	25.1(9.8);0.5	19.0(6.2);0.1	22.4(8.1);0.6	19.8(3.9);0.1	19.6(6.1);0.1	18.4(3.5);0.3	20.9(4.5);0.1	21.1(9.6);0.4
200W	Hip	1	0.938	0.521	2.917	20.418	25.9(5.9);0.0	25.9(5.9);0.0	25.9(5.9);0.0	26.8(7.4);0.2	22.6(7.1);0.3	25.3(8.0);0.0	26.1(6.5);0.0	24.7(8.6);0.3	25.3(6.0);0.0	25.3(6.2);0.0	26.0(6.2);0.1	24.7(5.8);0.2	26.4(7.5);0.0
200W	Hip	2	1.057	0.406	2.882	20.176	19.0(7.0);0.2	19.0(7.0);0.2	19.0(7.0);0.2	18.0(3.7);0.5	21.8(6.1);0.4	20.1(4.4);0.0	20.7(6.2);0.1	22.4(5.4);0.4	20.9(4.5);0.2	20.1(5.3);0.0	20.0(4.9);0.0	21.7(6.3);0.3	19.6(5.0);0.1
200W	Hip	3	0.933	0.527	3.123	21.859	21.4(6.4);0.0	21.4(6.4);0.0	21.4(6.4);0.0	22.2(7.6);0.1	19.9(6.7);0.1	21.9(8.2);0.0	22.7(8.0);0.0	22.0(8.0);0.0	21.8(7.0);0.0	21.2(7.1);0.0	22.0(7.1);0.1	21.7(8.1);0.1	22.2(7.9);0.1
200W	Hip	4	1.553	0.107	2.879	20.155	22.0(5.5);0.3	22.0(5.5);0.3	22.0(5.5);0.3	22.0(4.6);0.4	24.2(5.1);0.1	22.6(3.9);0.3	23.0(4.8);0.1	25.1(3.8);0.2	23.6(4.3);0.0	23.6(4.4);0.0	22.6(4.7);0.2	23.5(6.0);0.0	22.4(6.2);0.2

Mean Joint Moments ANOVA

Power	Joint	Phase	F value	p value	DoF1	DoF2	0lbs	2.5 lbs	5 lbs	7.5 lbs	10 lbs	12.5 lbs	15 lbs	17.5 lbs	20 lbs	22.5 lbs	25 lbs	27.5 lbs	30 lbs
100W	Ankle	1	20.421	<0.001	2.302	11.51	-0.0(0.0);1.9	-0.0(0.0);1.9	-0.0(0.0);1.9	-0.1(0.1);1.4	-0.1(0.1);1.1	-0.1(0.1);1.0	-0.1(0.1);0.9	-0.1(0.0);0.6	-0.1(0.1);0.7	-0.1(0.0);0.7	-0.1(0.1);0.6	-0.1(0.1);0.6	-0.1(0.1);0.5
100W	Ankle	2	8.86	<0.001	2.788	13.938	-0.2(0.1);0.7	-0.2(0.1);0.7	-0.2(0.1);0.7	-0.2(0.1);0.5	-0.2(0.1);0.4	-0.2(0.1);0.4	-0.2(0.1);0.3	-0.2(0.1);0.2	-0.2(0.1);0.2	-0.2(0.1);0.3	-0.2(0.1);0.2	-0.2(0.1);0.2	-0.2(0.1);0.0
100W	Ankle	3	20.89	<0.001	3.634	18.17	0.0(0.0);2.1	0.0(0.0);2.1	0.0(0.0);2.1	-0.0(0.0);1.5	-0.0(0.0);1.2	-0.0(0.0);0.8	-0.1(0.0);0.5	-0.1(0.0);0.6	-0.1(0.1);0.3	-0.1(0.0);0.6	-0.1(0.0);0.6	-0.1(0.0);0.3	-0.1(0.0);0.2
100W	Ankle	4	7.087	<0.001	2.547	12.736	0.0(0.0);1.9	0.0(0.0);1.9	0.0(0.0);1.9	-0.0(0.0);1.3	-0.0(0.0);1.0	-0.0(0.0);0.6	-0.0(0.0);1.0	-0.0(0.0);0.5	-0.0(0.0);0.7	-0.0(0.0);0.7	-0.0(0.0);0.7	-0.0(0.0);0.6	-0.0(0.0);0.4
100W	Knee	1	0.781	0.686	3.47	17.349	-0.1(0.1);0.2	-0.1(0.1);0.2	-0.1(0.1);0.2	-0.1(0.1);0.3	-0.1(0.1);0.1	-0.1(0.1);0.3	-0.1(0.1);0.2	-0.1(0.1);0.1	-0.0(0.1);0.4	-0.1(0.1);0.2	-0.1(0.1);0.2	-0.0(0.1);0.4	-0.0(0.1);0.5
100W	Knee	2	3.313	<0.001	2.026	10.131	-0.2(0.1);0.1	-0.2(0.1);0.1	-0.2(0.1);0.1	-0.2(0.2);0.1	-0.2(0.2);0.0	-0.1(0.3);0.2	-0.2(0.2);0.0	-0.2(0.2);0.1	-0.2(0.2);0.1	-0.2(0.3);0.0	-0.2(0.2);0.1	-0.2(0.3);0.0	-0.2(0.2);0.0
100W	Knee	3	7.105	<0.001	2.966	14.831	0.1(0.0);2.0	0.1(0.0);2.0	0.1(0.0);2.0	0.1(0.0);1.3	0.1(0.1);1.2	0.2(0.1);0.8	0.1(0.0);1.1	0.2(0.0);0.9	0.1(0.1);1.1	0.2(0.1);0.5	0.2(0.1);0.4	0.2(0.1);0.6	0.1(0.1);1.0
100W	Knee	4	8.471	<0.001	3.567	17.835	0.1(0.1);1.1	0.1(0.1);1.1	0.1(0.1);1.1	0.1(0.1);0.5	0.1(0.1);0.3	0.1(0.1);0.4	0.2(0.1);0.1	0.2(0.1);0.2	0.2(0.1);0.0	0.2(0.1);0.1	0.2(0.1);0.2	0.2(0.1);0.1	0.2(0.1);0.0
100W	Hip	1	8.442	<0.001	2.444	12.219	-0.2(0.2);1.3	-0.2(0.2);1.3	-0.2(0.2);1.3	-0.3(0.3);0.9	-0.4(0.3);0.6	-0.3(0.4);0.8	-0.5(0.3);0.5	-0.5(0.1);0.4	-0.4(0.3);0.4	-0.4(0.3);0.4	-0.5(0.2);0.3	-0.4(0.4);0.4	-0.4(0.3);0.4
100W	Hip	2	9.508	<0.001	2.35	11.75	-0.0(0.3);0.8	-0.0(0.3);0.8	-0.0(0.3);0.8	-0.1(0.2);0.6	-0.2(0.3);0.4	-0.1(0.4);0.4	-0.3(0.3);0.1	-0.2(0.3);0.2	-0.4(0.3);0.2	-0.2(0.4);0.2	-0.3(0.3);0.2	-0.3(0.3);0.1	-0.4(0.3);0.4
100W	Hip	3	2.015	0.029	2.128	10.64	0.6(0.1);1.1	0.6(0.1);1.1	0.6(0.1);1.1	0.6(0.2);0.8	0.5(0.2);0.7	0.4(0.3);0.2	0.4(0.2);0.4	0.5(0.1);0.6	0.4(0.2);0.3	0.5(0.2);0.4	0.5(0.1);0.7	0.4(0.3);0.2	0.4(0.2);0.1
100W	Hip	4	1.789	0.058	2.261	11.307	0.2(0.1);1.3	0.2(0.1);1.3	0.2(0.1);1.3	0.2(0.1);1.1	0.2(0.1);1.0	0.1(0.2);0.4	0.2(0.1);1.1	0.1(0.1);0.5	0.2(0.1);1.0	0.1(0.1);0.6	0.1(0.1);0.6	0.1(0.1);0.8	0.2(0.2);0.8
200W	Ankle	1	15.337	<0.001	2.924	20.471	-0.1(0.1);1.3	-0.1(0.1);1.3	-0.1(0.1);1.3	-0.2(0.1);1.1	-0.1(0.1);1.1	-0.2(0.1);0.9	-0.2(0.1);0.6	-0.2(0.1);0.5	-0.2(0.1);0.7	-0.2(0.1);0.6	-0.2(0.1);0.7	-0.2(0.1);0.8	-0.2(0.1);0.7
200W	Ankle	2	6.465	<0.001	2.853	19.973	-0.3(0.1);0.4	-0.3(0.1);0.4	-0.3(0.1);0.4	-0.3(0.1);0.7	-0.3(0.1);0.5	-0.3(0.1);0.4	-0.3(0.1);0.2	-0.3(0.1);0.3	-0.3(0.1);0.2	-0.3(0.1);0.3	-0.3(0.1);0.2	-0.3(0.1);0.2	-0.3(0.1);0.3
200W	Ankle	3	14.63	<0.001	3.829	26.801	0.0(0.0);2.1	0.0(0.0);2.1	0.0(0.0);2.1	-0.0(0.0);1.5	-0.0(0.0);1.3	-0.0(0.0);1.2	-0.0(0.0);0.8	-0.0(0.0);0.8	-0.0(0.0);0.9	-0.0(0.0);1.1	-0.0(0.0);1.0	-0.0(0.0);0.8	-0.0(0.0);0.6
200W	Ankle	4	9.496	<0.001	2.76	19.32	0.0(0.0);2.0	0.0(0.0);2.0	0.0(0.0);2.0	-0.0(0.0);1.4	-0.0(0.0);1.5	-0.0(0.0);1.2	-0.0(0.0);1.1	-0.0(0.0);0.9	-0.0(0.0);1.4	-0.0(0.0);1.0	-0.0(0.0);1.2	-0.0(0.0);1.3	-0.0(0.0);0.8
200W	Knee	1	0.66	0.807	3.111	21.776	-0.1(0.2);0.1	-0.1(0.2);0.1	-0.1(0.2);0.1	-0.1(0.1);0.1	-0.0(0.2);0.1	-0.1(0.2);0.1	-0.1(0.2);0.1	-0.1(0.2);0.0	-0.0(0.2);0.2	-0.1(0.1);0.0	-0.0(0.2);0.2	-0.0(0.2);0.4	-0.0(0.2);0.2
200W	Knee	2	1.459	0.141	2.854	19.977	-0.3(0.2);0.0	-0.3(0.2);0.0	-0.3(0.2);0.0	-0.1(0.3);0.4	-0.2(0.3);0.1	-0.2(0.3);0.1	-0.3(0.3);0.1	-0.3(0.4);0.1	-0.3(0.3);0.0	-0.3(0.3);0.1	-0.2(0.4);0.1	-0.3(0.3);0.0	-0.1(0.3);0.5
200W	Knee	3	6.973	<0.001	3.455	24.188	0.1(0.1);1.1	0.1(0.1);1.1	0.1(0.1);1.1	0.1(0.1);0.7	0.1(0.0);0.9	0.1(0.1);0.6	0.1(0.1);0.8	0.1(0.0);0.8	0.1(0.1);0.8	0.1(0.1);0.6	0.1(0.1);0.8	0.1(0.1);0.9	0.1(0.1);0.8
200W	Knee	4	8.306	<0.001	3.834	26.84	0.1(0.1);1.0	0.1(0.1);1.0	0.1(0.1);1.0	0.1(0.1);0.8	0.1(0.1);0.6	0.1(0.1);0.5	0.1(0.1);0.2	0.1(0.1);0.6	0.1(0.1);0.3	0.1(0.1);0.2	0.1(0.1);0.3	0.1(0.1);0.2	0.1(0.1);0.9
200W	Hip	1	8.715	<0.001	3.231	22.615	-0.6(0.3);0.9	-0.6(0.3);0.9	-0.6(0.3);0.9	-0.5(0.4);1.0	-0.6(0.4);0.7	-0.7(0.3);0.8	-0.8(0.3);0.4	-0.8(0.4);0.5	-0.8(0.4);0.4	-0.8(0.3);0.4	-0.7(0.5);0.5	-0.7(0.4);0.5	-0.6(0.5);0.8
200W	Hip	2	4.269	<0.001	2.976	20.834	-0.4(0.3);0.5	-0.4(0.3);0.5	-0.4(0.3);0.5	-0.3(0.3);0.7	-0.4(0.3);0.2	-0.5(0.5);0.3	-0.6(0.4);0.1	-0.5(0.5);0.3	-0.6(0.3);0.1	-0.5(0.3);0.2	-0.5(0.3);0.2	-0.6(0.3);0.0	-0.5(0.3);0.5
200W	Hip	3	4.424	<0.001	2.178	15.245	0.6(0.1);1.3	0.6(0.1);1.3	0.6(0.1);1.3	0.5(0.3);0.3	0.5(0.3);0.5	0.5(0.2);0.6	0.5(0.2);0.6	0.5(0.3);0.3	0.5(0.2);0.5	0.5(0.2);0.7	0.5(0.3);0.4	0.5(0.3);0.4	0.4(0.3);0.0
200W	Hip	4	2.807	0.001	2.521	17.646	0.2(0.1);1.4	0.2(0.1);1.4	0.2(0.1);1.4	0.1(0.2);0.7	0.2(0.1);1.0	0.1(0.2);0.4	0.2(0.1);0.9	0.1(0.1);0.7	0.2(0.1);1.0	0.1(0.1);0.7	0.2(0.1);0.8	0.2(0.2);1.2	0.2(0.2);0.6

Peak to Peak Joint Moments ANOVA

Power	Joint	Phase	F value	p value	DoF1	DoF2	0lbs	2.5 lbs	5 lbs	7.5 lbs	10 lbs	12.5 lbs	15 lbs	17.5 lbs	20 lbs	22.5 lbs	25 lbs	27.5 lbs	30 lbs
100W	Ankle	1	1.866	0.046	2.346	11.728	0.2(0.1);0.3	0.2(0.1);0.3	0.2(0.1);0.3	0.2(0.1);0.3	0.2(0.1);0.3	0.2(0.1);0.3	0.2(0.1);0.3	0.2(0.1);0.0	0.2(0.1);0.2	0.2(0.1);0.3	0.2(0.1);0.2	0.2(0.1);0.2	0.2(0.1);0.2
100W	Ankle	2	1.244	0.265	2.332	11.662	0.2(0.1);0.2	0.2(0.1);0.2	0.2(0.1);0.2	0.1(0.1);0.1	0.1(0.1);0.1	0.1(0.1);0.2	0.1(0.1);0.3	0.1(0.0);0.2	0.1(0.1);0.2	0.1(0.1);0.4	0.1(0.1);0.2	0.1(0.1);0.3	0.1(0.0);0.4
100W	Ankle	3	1.478	0.143	3.22	16.101	0.2(0.1);0.6	0.2(0.1);0.6	0.2(0.1);0.6	0.1(0.1);0.5	0.1(0.1);0.4	0.1(0.1);0.2	0.2(0.1);0.5	0.1(0.1);0.4	0.1(0.1);0.5	0.1(0.1);0.4	0.2(0.1);0.5	0.1(0.1);0.5	0.2(0.1);0.8
100W	Ankle	4	1.189	0.304	2.046	10.229	0.0(0.0);0.1	0.0(0.0);0.1	0.0(0.0);0.1	0.0(0.0);0.4	0.0(0.0);0.4	0.0(0.0);0.3	0.0(0.0);0.1	0.0(0.0);0.8	0.0(0.0);0.4	0.0(0.0);0.4	0.0(0.0);0.4	0.0(0.0);0.3	0.0(0.0);0.2
100W	Knee	1	0.98	0.482	2.245	11.226	0.4(0.1);0.2	0.4(0.1);0.2	0.4(0.1);0.2	0.4(0.2);0.0	0.4(0.2);0.0	0.3(0.2);0.1	0.4(0.2);0.2	0.4(0.2);0.1	0.4(0.1);0.1	0.4(0.2);0.1	0.4(0.2);0.1	0.4(0.1);0.1	0.3(0.1);0.1
100W	Knee	2	1.141	0.34	3.724	18.618	0.4(0.1);0.2	0.4(0.1);0.2	0.4(0.1);0.2	0.4(0.2);0.2	0.4(0.1);0.1	0.4(0.1);0.0	0.4(0.1);0.2	0.4(0.1);0.1	0.3(0.1);0.2	0.3(0.1);0.2	0.4(0.1);0.1	0.3(0.1);0.2	0.4(0.2);0.1
100W	Knee	3	3.107	<0.001	1.542	7.709	0.2(0.1);0.4	0.2(0.1);0.4	0.2(0.1);0.4	0.2(0.1);0.7	0.2(0.1);0.6	0.3(0.2);0.2	0.3(0.2);0.2	0.2(0.2);0.3	0.3(0.2);0.1	0.3(0.2);0.1	0.3(0.2);0.3	0.3(0.2);0.0	0.3(0.1);0.1
100W	Knee	4	1.464	0.148	2.766	13.829	0.2(0.0);0.5	0.2(0.0);0.5	0.2(0.0);0.5	0.1(0.1);0.8	0.2(0.1);0.6	0.2(0.1);0.4	0.2(0.1);0.5	0.2(0.1);0.1	0.2(0.1);0.4	0.2(0.1);0.4	0.2(0.1);0.1	0.2(0.1);0.2	0.2(0.1);0.2
100W	Hip	1	1.242	0.267	2.459	12.296	0.6(0.2);0.1	0.6(0.2);0.1	0.6(0.2);0.1	0.6(0.3);0.0	0.6(0.3);0.1	0.6(0.3);0.1	0.6(0.3);0.1	0.6(0.2);0.1	0.7(0.3);0.2	0.6(0.2);0.1	0.6(0.2);0.0	0.6(0.2);0.1	0.6(0.2);0.1
100W	Hip	2	0.586	0.867	2.798	13.992	1.3(0.3);0.3	1.3(0.3);0.3	1.3(0.3);0.3	1.2(0.4);0.1	1.2(0.3);0.2	1.1(0.5);0.1	1.2(0.3);0.1	1.3(0.3);0.2	1.2(0.3);0.0	1.1(0.3);0.0	1.3(0.4);0.2	1.2(0.3);0.2	1.2(0.3);0.0
100W	Hip	3	2.072	0.024	2.635	13.173	0.5(0.2);0.8	0.5(0.2);0.8	0.5(0.2);0.8	0.4(0.2);0.2	0.3(0.2);0.0	0.4(0.2);0.5	0.5(0.2);0.5	0.3(0.2);0.1	0.4(0.3);0.3	0.4(0.2);0.2	0.4(0.2);0.1	0.4(0.2);0.3	0.5(0.3);0.8
100W	Hip	4	8.697	<0.001	2.132	10.66	0.5(0.1);1.4	0.5(0.1);1.4	0.5(0.1);1.4	0.6(0.1);0.5	0.7(0.2);0.3	0.6(0.2);0.5	0.7(0.2);0.3	0.7(0.2);0.1	0.7(0.1);0.3	0.7(0.2);0.0	0.7(0.2);0.0	0.7(0.2);0.0	0.7(0.2);0.2
200W	Ankle	1	3.329	<0.001	2.606	18.241	0.3(0.1);0.0	0.3(0.1);0.0	0.3(0.1);0.0	0.3(0.1);0.3	0.3(0.1);0.3	0.3(0.1);0.2	0.3(0.1);0.0	0.3(0.1);0.0	0.3(0.1);0.1	0.3(0.1);0.0	0.3(0.1);0.1	0.3(0.1);0.0	0.3(0.1);0.3
200W	Ankle	2	2.693	0.002	1.892	13.242	0.3(0.1);0.2	0.3(0.1);0.2	0.3(0.1);0.2	0.2(0.1);0.4	0.2(0.1);0.6	0.2(0.1);0.3	0.2(0.1);0.4	0.2(0.1);0.4	0.2(0.1);0.4	0.3(0.1);0.3	0.2(0.1);0.4	0.2(0.1);0.4	0.2(0.1);0.5
200W	Ankle	3	1.106	0.363	2.466	17.259	0.2(0.1);0.6	0.2(0.1);0.6	0.2(0.1);0.6	0.2(0.1);0.3	0.2(0.1);0.6	0.2(0.1);0.3	0.2(0.1);0.5	0.2(0.1);0.4	0.2(0.1);0.6	0.2(0.1);0.5	0.2(0.1);0.6	0.2(0.1);0.6	0.2(0.1);0.5
200W	Ankle	4	0.545	0.9	3.2	22.402	0.1(0.0);0.5	0.1(0.0);0.5	0.1(0.0);0.5	0.1(0.0);0.4	0.1(0.0);0.3	0.1(0.0);0.6	0.1(0.0);0.4	0.1(0.0);0.4	0.1(0.0);0.5	0.1(0.0);0.4	0.1(0.0);0.5	0.1(0.0);0.6	0.1(0.0);0.3
200W	Knee	1	2.028	0.023	2.287	16.006	0.5(0.2);0.2	0.5(0.2);0.2	0.5(0.2);0.2	0.4(0.1);0.4	0.4(0.1);0.1	0.4(0.2);0.2	0.5(0.2);0.1	0.5(0.2);0.0	0.5(0.2);0.0	0.5(0.2);0.0	0.5(0.2);0.0	0.5(0.2);0.1	0.5(0.1);0.2
200W	Knee	2	0.843	0.622	2.964	20.747	0.5(0.2);0.1	0.5(0.2);0.1	0.5(0.2);0.1	0.6(0.2);0.2	0.4(0.2);0.1	0.5(0.2);0.1	0.5(0.2);0.0	0.5(0.2);0.0	0.5(0.2);0.0	0.5(0.2);0.0	0.5(0.2);0.1	0.5(0.2);0.1	0.5(0.2);0.2
200W	Knee	3	1.073	0.391	1.991	13.939	0.3(0.1);0.0	0.3(0.1);0.0	0.3(0.1);0.0	0.3(0.2);0.1	0.3(0.2);0.1	0.3(0.2);0.0	0.3(0.2);0.1	0.3(0.2);0.2	0.3(0.2);0.1	0.3(0.2);0.1	0.3(0.2);0.1	0.3(0.2);0.1	0.2(0.1);0.0
200W	Knee	4	3.93	<0.001	3.508	24.553	0.2(0.1);0.2	0.2(0.1);0.2	0.2(0.1);0.2	0.2(0.1);0.1	0.2(0.1);0.2	0.2(0.1);0.2	0.2(0.1);0.3	0.2(0.1);0.0	0.2(0.1);0.4	0.2(0.1);0.3	0.2(0.1);0.3	0.2(0.1);0.2	0.2(0.1);0.2
200W	Hip	1	2.072	0.02	3.468	24.277	1.0(0.4);0.1	1.0(0.4);0.1	1.0(0.4);0.1	0.8(0.2);0.4	0.9(0.3);0.1	1.0(0.3);0.0	1.0(0.4);0.0	1.0(0.4);0.0	1.1(0.3);0.2	1.0(0.3);0.0	1.0(0.3);0.0	1.1(0.3);0.2	0.9(0.3);0.3
200W	Hip	2	0.682	0.786	2.342	16.397	1.8(0.5);0.2	1.8(0.5);0.2	1.8(0.5);0.2	1.6(0.6);0.1	1.6(0.6);0.1	1.6(0.6);0.0	1.8(0.4);0.2	1.7(0.5);0.0	1.8(0.4);0.3	1.8(0.5);0.2	1.8(0.4);0.2	1.7(0.4);0.1	1.6(0.5);0.2
200W	Hip	3	2.111	0.017	2.449	17.144	0.4(0.2);0.4	0.4(0.2);0.4	0.4(0.2);0.4	0.5(0.4);0.4	0.4(0.2);0.4	0.5(0.2);0.7	0.4(0.3);0.2	0.4(0.2);0.6	0.4(0.3);0.2	0.4(0.2);0.1	0.5(0.3);0.4	0.5(0.4);0.4	0.5(0.3);0.7
200W	Hip	4	3.265	<0.001	2.338	16.368	0.7(0.2);0.5	0.7(0.2);0.5	0.7(0.2);0.5	0.8(0.3);0.3	0.8(0.3);0.2	0.7(0.2);0.5	0.8(0.2);0.2	0.8(0.2);0.3	0.8(0.3);0.2	0.9(0.2);0.0	0.8(0.2);0.2	0.8(0.2);0.3	0.8(0.3);0.4

Mean Pedal Data ANOVA

Power	Joint	Phase	F value	p value	DoF1	DoF2	0lbs	2.5 lbs	5 lbs	7.5 lbs	10 lbs	12.5 lbs	15 lbs	17.5 lbs	20 lbs	22.5 lbs	25 lbs	27.5 lbs	30 lbs
100W	Ankle	1	45.083	<0.001	3.512	24.584	42.3(10.3);4.2	42.3(10.3);4.2	42.3(10.3);4.2	60.3(13.0);2.6	67.6(11.0);2.3	75.5(8.1);1.9	79.1(10.1);1.5	82.0(6.9);1.4	81.6(12.4);1.2	82.0(13.2);1.2	84.9(11.9);1.0	90.1(13.0);0.7	91.0(13.2);0.6
100W	Ankle	2	54.047	<0.001	3.456	24.19	66.1(13.4);2.4	66.1(13.4);2.4	66.1(13.4);2.4	79.7(12.8);1.7	86.3(17.3);1.1	87.2(17.2);1.0	90.3(14.3);1.0	90.6(19.7);0.7	93.5(15.7);0.8	92.5(19.5);0.7	95.9(16.6);0.6	96.9(17.4);0.6	99.8(13.7);0.4
100W	Ankle	3	44.703	<0.001	3.671	25.698	2.6(11.6);4.2	2.6(11.6);4.2	2.6(11.6);4.2	-12.4(17.5);2.4	-18.8(15.8);2.1	-25.2(15.9);1.6	-30.3(16.6);1.3	-30.8(12.2);1.4	-36.6(17.6);0.9	-32.3(16.3);1.2	-34.4(15.2);1.1	-38.3(13.5);0.9	-38.0(12.0);1.0
100W	Ankle	4	51.859	<0.001	3.658	25.606	5.1(8.8);4.1	5.1(8.8);4.1	5.1(8.8);4.1	-13.8(10.3);2.3	-20.8(11.1);1.6	-23.2(14.8);1.2	-26.7(10.4);1.2	-28.3(16.2);0.7	-31.6(17.6);0.6	-29.0(15.9);0.8	-31.8(12.6);0.7	-32.1(13.2);0.7	-33.8(10.4);0.6
100W	Knee	1	23.677	<0.001	3.458	24.208	-20.2(11.9);2.7	-20.2(11.9);2.7	-20.2(11.9);2.7	-9.1(10.3);2.0	-6.5(10.4);1.7	-5.3(10.3);1.5	-1.8(11.9);1.2	-3.9(8.9);1.4	2.6(10.9);0.9	1.5(10.3);1.0	4.5(12.0);0.7	7.1(11.7);0.5	9.4(11.7);0.3
100W	Knee	2	10.573	<0.001	3.668	25.676	-121.0(43.2);0.9	-121.0(43.2);0.9	-121.0(43.2);0.9	-130.1(42.2);0.7	-135.1(41.8);0.6	-135.9(39.2);0.5	-140.9(38.6);0.4	-141.4(40.7);0.3	-143.6(35.3);0.4	-141.8(35.8);0.4	-150.8(35.7);0.2	-148.1(36.8);0.3	-151.9(31.6);0.2
100W	Knee	3	42.464	<0.001	4.239	29.674	-34.5(34.4);2.1	-34.5(34.4);2.1	-34.5(34.4);2.1	-55.3(27.9);1.7	-63.3(30.9);1.2	-66.4(29.4);1.1	-77.0(32.1);0.7	-73.7(29.5);0.7	-84.1(32.7);0.4	-79.7(27.3);0.7	-83.0(30.9);0.5	-87.9(27.8);0.4	-90.5(22.6);0.3
100W	Knee	4	2.088	0.019	1.936	13.555	14.6(12.3);0.9	14.6(12.3);0.9	14.6(12.3);0.9	19.6(8.3);0.6	20.3(7.9);0.5	23.4(6.3);0.2	20.2(10.2);0.5	20.8(7.2);0.5	22.3(9.1);0.3	23.8(12.3);0.1	24.3(10.7);0.0	24.5(10.4);0.0	26.6(14.0);0.2
100W	Hip	1	3.505	<0.001	3.805	26.632	-3.6(6.5);0.9	-3.6(6.5);0.9	-3.6(6.5);0.9	-4.4(6.4);0.8	-4.9(6.8);0.6	-5.2(6.3);0.6	-4.5(6.5);0.7	-4.2(6.6);0.8	-4.5(6.1);0.8	-3.9(5.5);0.9	-4.0(6.3);0.8	-4.7(7.0);0.7	-4.2(6.4);0.8
100W	Hip	2	3.816	<0.001	2.511	17.578	-3.6(4.8);0.9	-3.6(4.8);0.9	-3.6(4.8);0.9	-4.7(5.6);0.7	-5.3(6.2);0.5	-5.5(6.3);0.5	-5.9(4.9);0.5	-6.4(6.0);0.3	-6.0(5.4);0.5	-5.5(5.2);0.5	-6.7(5.3);0.3	-6.9(6.1);0.3	-6.8(5.9);0.3
100W	Hip	3	0.699	0.771	1.868	13.075	-0.2(7.2);0.5	-0.2(7.2);0.5	-0.2(7.2);0.5	0.3(6.2);0.4	1.0(6.1);0.3	0.3(7.6);0.3	0.3(5.9);0.4	-0.4(7.2);0.5	-0.2(5.4);0.5	1.2(4.8);0.3	0.3(5.6);0.4	0.6(4.1);0.4	0.3(5.0);0.5
100W	Hip	4	1.231	0.266	2.525	17.676	1.3(4.4);0.2	1.3(4.4);0.2	1.3(4.4);0.2	1.3(4.1);0.2	1.8(4.4);0.1	1.3(5.0);0.2	2.4(4.9);0.0	1.9(4.3);0.0	1.4(3.7);0.2	2.4(4.2);0.0	2.8(4.1);0.1	2.7(3.9);0.1	2.7(3.9);0.1
200W	Ankle	1	29.449	<0.001	3.317	23.219	109.6(16.4);3.2	109.6(16.4);3.2	109.6(16.4);3.2	126.9(17.1);2.2	130.4(12.3);2.2	137.7(11.0);1.9	143.6(13.2);1.5	142.0(11.9);1.5	143.2(12.1);1.5	143.0(14.2);1.4	141.5(12.6);1.6	141.5(14.1);1.5	141.9(16.1);1.3
200W	Ankle	2	22.42	<0.001	3.148	22.036	116.9(19.0);1.8	116.9(19.0);1.8	116.9(19.0);1.8	125.3(20.0);1.4	128.3(20.4);1.2	133.8(19.2);1.0	135.4(20.8);0.9	131.0(22.0);0.9	137.8(20.7);0.8	136.1(21.3);0.9	139.2(18.6);0.8	136.9(24.8);0.8	136.3(21.3);0.8
200W	Ankle	3	35.925	<0.001	3.099	21.691	11.5(11.6);3.4	11.5(11.6);3.4	11.5(11.6);3.4	0.1(18.8);2.1	-5.2(12.6);2.1	-10.5(17.4);1.6	-15.3(22.3);1.1	-12.4(14.8);1.5	-15.4(15.6);1.4	-13.0(15.2);1.6	-14.8(17.5);1.4	-14.5(16.2);1.4	-14.2(18.0);1.3
200W	Ankle	4	26.296	<0.001	2.392	16.745	10.5(11.9);2.9	10.5(11.9);2.9	10.5(11.9);2.9	-4.5(12.6);1.9	-5.4(11.7);1.8	-11.1(15.8);1.4	-13.7(17.8);1.1	-12.7(19.4);1.0	-15.1(15.2);1.2	-15.9(14.6);1.1	-16.8(12.6);1.2	-14.0(18.6);1.1	-15.2(17.9);1.0
200W	Knee	1	23.016	<0.001	2.461	17.226	-17.5(16.2);2.9	-17.5(16.2);2.9	-17.5(16.2);2.9	-2.0(13.1);2.2	0.9(17.2);1.7	6.8(13.0);1.6	9.5(15.5);1.3	9.0(13.1);1.3	10.4(15.9);1.2	10.2(14.0);1.3	11.6(15.7);1.2	8.5(12.6);1.4	12.3(12.1);1.2
200W	Knee	2	8.532	<0.001	3.241	22.687	-188.1(48.8);0.6	-188.1(48.8);0.6	-188.1(48.8);0.6	-190.8(54.9);0.6	-191.0(38.9);0.5	-198.6(42.4);0.5	-200.8(54.9);0.4	-195.2(47.4);0.3	-205.4(46.6);0.3	-200.0(43.6);0.4	-204.9(43.4);0.3	-203.2(53.5);0.4	-198.9(51.2);0.3
200W	Knee	3	22.577	<0.001	3.28	22.957	-35.7(35.0);1.9	-35.7(35.0);1.9	-35.7(35.0);1.9	-46.1(36.3);1.5	-50.9(32.7);1.3	-59.5(34.0);1.1	-63.8(40.6);0.8	-56.3(33.1);1.1	-62.8(33.8);1.0	-60.3(31.8);1.1	-63.3(34.0);1.0	-64.0(37.8);0.9	-61.6(36.9);0.9
200W	Knee	4	3.035	<0.001	2.67	18.688	15.1(13.2);1.2	15.1(13.2);1.2	15.1(13.2);1.2	23.8(10.3);0.5	23.5(10.2);0.5	24.3(8.9);0.5	25.3(10.5);0.4	23.1(8.3);0.6	24.5(11.2);0.4	26.4(14.7);0.2	25.8(11.8);0.3	25.3(11.9);0.3	25.9(13.0);0.2
200W	Hip	1	4.542	<0.001	3.317	23.218	-8.9(10.6);1.1	-8.9(10.6);1.1	-8.9(10.6);1.1	-11.4(10.1);0.9	-12.4(10.7);0.7	-11.2(10.4);0.9	-12.3(10.3);0.7	-11.3(9.7);1.0	-11.3(10.3);0.9	-12.0(8.6);0.9	-11.4(9.7);0.9	-11.4(8.0);1.0	-11.5(9.1);0.9
200W	Hip	2	5.784	<0.001	2.879	20.155	-11.5(6.9);1.0	-11.5(6.9);1.0	-11.5(6.9);1.0	-12.9(8.6);0.7	-13.1(8.1);0.6	-13.3(7.8);0.7	-13.8(8.3);0.6	-13.8(8.7);0.6	-14.6(9.0);0.5	-14.5(8.6);0.6	-15.3(8.3);0.5	-15.0(7.3);0.5	-14.2(8.6);0.5
200W	Hip	3	1.219	0.274	1.665	11.655	-0.2(9.0);0.7	-0.2(9.0);0.7	-0.2(9.0);0.7	0.1(8.1);0.7	-0.3(6.9);0.8	1.1(7.7);0.6	0.1(7.5);0.7	-0.2(8.3);0.7	0.1(7.1);0.7	0.5(6.7);0.7	-0.1(7.2);0.8	0.5(7.2);0.7	-0.2(8.2);0.7
200W	Hip	4	1.059	0.403	2.055	14.385	1.9(5.1);0.3	1.9(5.1);0.3	1.9(5.1);0.3	1.9(5.0);0.3	1.6(4.6);0.4	2.4(5.2);0.2	2.5(4.9);0.2	2.2(5.4);0.2	2.5(4.7);0.2	2.9(4.6);0.1	2.8(4.6);0.1	3.0(5.3);0.1	1.6(5.4);0.3

Peak to Peak Pedal Data ANOVA

Power	Joint	Phase	F value	p value	DoF1	DoF2	0lbs	2.5 lbs	5 lbs	7.5 lbs	10 lbs	12.5 lbs	15 lbs	17.5 lbs	20 lbs	22.5 lbs	25 lbs	27.5 lbs	30 lbs
100W	Ankle	1	22.814	<0.001	3.546	24.822	109.4(22.2);2.0	109.4(22.2);2.0	109.4(22.2);2.0	111.4(29.6);1.8	122.3(27.2);1.5	127.7(27.2);1.3	132.7(20.2);1.3	136.5(26.1);1.0	138.2(24.5);1.1	132.7(28.5);1.2	141.0(23.9);1.1	147.1(22.9);0.9	149.0(22.3);0.8
100W	Ankle	2	17.875	<0.001	3.182	22.276	111.1(31.7);1.8	111.1(31.7);1.8	111.1(31.7);1.8	122.5(44.1);1.3	131.1(40.8);1.2	140.9(36.1);1.0	142.3(31.4);1.0	149.2(23.8);0.8	147.6(28.8);0.9	139.8(35.9);1.0	147.9(25.2);0.9	153.0(25.8);0.8	153.4(23.3);0.8
100W	Ankle	3	22.87	<0.001	2.718	19.024	29.6(11.9);4.6	29.6(11.9);4.6	29.6(11.9);4.6	46.7(16.8);2.6	55.2(17.8);2.0	59.9(23.2);1.3	62.1(17.6);1.6	63.0(20.9);1.2	68.2(25.3);0.9	69.1(20.1);1.0	72.9(22.6);0.7	76.6(20.7);0.6	79.1(15.6);0.5
100W	Ankle	4	67.48	<0.001	3.264	22.849	17.3(7.6);4.9	17.3(7.6);4.9	17.3(7.6);4.9	49.8(16.1);2.3	60.2(15.3);1.7	70.9(17.3);1.1	71.7(17.2);1.1	76.7(16.2);0.7	78.4(17.9);0.7	78.6(18.9);0.7	83.1(22.1);0.4	84.4(22.0);0.4	87.4(20.0);0.2
100W	Knee	1	6.299	<0.001	2.693	18.849	94.5(24.1);1.6	94.5(24.1);1.6	94.5(24.1);1.6	101.4(33.2);1.2	106.7(27.6);1.2	114.3(27.3);1.0	112.6(21.6);1.1	110.4(18.2);1.1	114.0(24.6);1.0	111.9(27.0);1.0	114.1(26.3);0.9	115.6(26.9);0.9	113.5(22.9);1.0
100W	Knee	2	1.827	0.045	2.739	19.173	105.4(48.1);0.3	105.4(48.1);0.3	105.4(48.1);0.3	96.6(35.1);0.2	96.4(34.5);0.1	89.8(24.3);0.1	95.1(32.3);0.1	93.7(29.3);0.2	102.8(31.6);0.3	99.2(32.0);0.2	116.8(35.0);0.7	114.1(43.6);0.6	124.7(38.7);0.9
100W	Knee	3	2.962	<0.001	4.31	30.173	131.6(58.9);0.1	131.6(58.9);0.1	131.6(58.9);0.1	127.0(46.4);0.2	127.8(47.8);0.2	124.8(42.3);0.3	133.3(50.3);0.1	134.3(42.9);0.0	140.6(43.3);0.1	136.8(36.0);0.0	151.1(41.0);0.3	148.1(45.3);0.3	159.6(38.7);0.6
100W	Knee	4	9.4	<0.001	3.273	22.913	34.8(9.4);2.3	34.8(9.4);2.3	34.8(9.4);2.3	46.4(13.8);1.6	51.0(17.0);1.3	56.4(17.0);1.0	56.8(15.9);1.1	54.4(15.7);1.0	61.8(18.9);0.8	59.0(18.3);1.0	62.0(19.7);0.8	66.4(23.0);0.6	65.5(15.6);0.7
100W	Hip	1	3.364	<0.001	2.047	14.327	14.8(5.8);0.5	14.8(5.8);0.5	14.8(5.8);0.5	12.4(3.5);1.2	12.5(3.9);1.1	13.8(4.7);1.0	13.2(3.7);1.0	13.1(3.3);1.2	12.7(4.2);1.1	12.3(3.5);1.3	13.6(3.9);0.9	13.7(4.2);0.8	12.9(3.2);1.1
100W	Hip	2	2.171	0.014	1.86	13.02	16.1(10.9);0.1	16.1(10.9);0.1	16.1(10.9);0.1	14.3(8.0);0.4	13.6(7.1);0.6	15.9(7.7);0.3	13.9(7.8);0.5	14.4(9.6);0.4	12.9(7.0);0.7	12.8(4.9);0.9	14.8(6.7);0.4	13.8(4.6);0.7	12.3(5.9);0.9
100W	Hip	3	4.006	<0.001	1.985	13.895	12.9(6.4);1.1	12.9(6.4);1.1	12.9(6.4);1.1	10.6(4.4);0.9	10.1(3.7);0.8	10.3(4.9);0.8	9.6(4.8);0.6	11.9(4.8);1.2	10.7(4.2);0.9	10.3(4.6);0.8	11.5(5.6);1.0	12.4(6.0);1.1	12.3(6.1);1.0
100W	Hip	4	5.737	<0.001	3.802	26.613	8.0(2.8);0.7	8.0(2.8);0.7	8.0(2.8);0.7	7.0(3.2);0.4	6.8(3.2);0.3	6.2(3.3);0.2	5.6(2.0);0.1	5.7(2.9);0.0	5.7(2.9);0.0	5.5(1.5);0.2	5.6(2.4);0.1	6.3(2.8);0.2	5.8(2.3);0.0
200W	Ankle	1	17.918	<0.001	3.064	21.449	194.6(27.0);1.8	194.6(27.0);1.8	194.6(27.0);1.8	203.5(36.1);1.4	203.3(20.7);1.6	218.9(22.4);1.2	228.1(30.9);1.0	223.2(32.2);0.9	233.4(29.2);0.8	223.5(29.7);1.1	231.6(22.6);0.9	225.0(29.5);1.1	224.3(25.2);1.0
200W	Ankle	2	15.997	<0.001	2.971	20.798	205.3(33.6);1.6	205.3(33.6);1.6	205.3(33.6);1.6	218.4(47.4);1.2	220.8(24.8);1.2	235.1(32.7);1.0	244.7(44.7);0.7	242.6(33.7);0.7	250.0(36.7);0.6	239.9(36.9);0.8	247.7(31.9);0.7	244.0(36.0);0.8	241.8(32.9);0.7
200W	Ankle	3	5.594	<0.001	3.292	23.042	40.5(12.3);2.4	40.5(12.3);2.4	40.5(12.3);2.4	46.5(14.8);1.8	51.9(17.1);1.3	50.9(14.7);1.5	51.6(16.0);1.4	50.3(16.3);1.4	47.7(12.3);1.9	49.8(17.9);1.4	52.1(14.5);1.5	52.3(15.8);1.3	51.8(16.7);1.4
200W	Ankle	4	26.535	<0.001	3.526	24.68	29.7(9.6);3.4	29.7(9.6);3.4	29.7(9.6);3.4	54.3(17.6);1.7	60.4(18.0);1.3	63.8(17.3);1.2	66.4(22.9);1.0	67.7(22.1);0.9	63.9(17.9);1.2	68.8(20.1);0.9	66.9(20.7);1.0	68.7(21.8);0.9	66.6(25.3);0.9
200W	Knee	1	10.867	<0.001	3.199	22.394	152.8(30.2);1.8	152.8(30.2);1.8	152.8(30.2);1.8	169.7(35.3);1.3	166.4(22.9);1.4	173.9(20.5);1.4	181.2(31.0);1.0	174.4(24.9);1.2	181.4(26.6);1.1	180.5(25.1);1.2	181.2(25.3);1.1	175.8(25.1);1.2	177.1(27.2);1.1
200W	Knee	2	0.407	0.97	2.858	20.009	118.2(43.5);0.1	118.2(43.5);0.1	118.2(43.5);0.1	112.4(42.4);0.2	108.6(38.3);0.2	114.3(32.9);0.2	116.4(39.7);0.2	109.1(36.7);0.1	113.6(38.9);0.2	109.2(36.9);0.3	114.7(37.0);0.2	119.5(45.8);0.1	116.5(43.7);0.1
200W	Knee	3	0.948	0.511	2.427	16.989	161.0(61.1);0.1	161.0(61.1);0.1	161.0(61.1);0.1	157.4(56.0);0.2	157.8(46.4);0.1	162.2(48.0);0.1	168.3(62.9);0.0	162.0(56.1);0.1	170.4(53.7);0.1	161.7(41.4);0.1	171.3(48.0);0.1	171.7(57.3);0.1	165.7(58.8);0.1
200W	Knee	4	11.198	<0.001	3.644	25.508	39.1(14.7);2.5	39.1(14.7);2.5	39.1(14.7);2.5	52.6(20.4);1.9	50.4(13.5);2.1	58.7(16.4);1.7	60.7(22.1);1.5	57.5(22.2);1.4	58.3(16.5);1.7	62.4(16.4);1.6	61.8(20.9);1.5	59.3(23.7);1.5	60.1(24.0);1.4
200W	Hip	1	5.584	<0.001	2.338	16.363	21.8(6.7);1.3	21.8(6.7);1.3	21.8(6.7);1.3	22.4(6.9);1.2	22.6(7.1);1.1	22.9(5.9);1.2	24.9(6.8);0.9	23.5(6.2);1.4	24.9(6.5);0.9	24.8(7.4);0.9	25.3(7.0);0.8	24.8(8.0);0.8	23.1(7.6);1.0
200W	Hip	2	2.18	0.014	1.486	10.405	22.4(11.8);0.8	22.4(11.8);0.8	22.4(11.8);0.8	22.5(7.9);1.0	22.7(8.5);0.9	23.4(8.2);0.9	24.2(8.4);0.8	21.5(7.7);1.3	22.7(8.6);0.9	22.6(6.9);1.0	23.7(8.6);0.8	22.9(5.5);1.1	22.4(7.9);0.9
200W	Hip	3	1.31	0.216	3.139	21.972	13.4(4.6);0.1	13.4(4.6);0.1	13.4(4.6);0.1	14.4(4.8);0.3	13.8(4.6);0.3	13.5(3.7);0.2	12.9(5.7);0.1	15.6(4.7);0.8	14.9(6.4);0.4	14.0(5.3);0.2	14.9(5.4);0.4	13.8(5.0);0.3	14.3(5.3);0.4
200W	Hip	4	3.351	<0.001	3.229	22.604	11.1(4.3);0.4	11.1(4.3);0.4	11.1(4.3);0.4	10.1(3.9);0.1	10.1(3.9);0.1	9.7(3.9);0.0	8.8(3.4);0.2	8.9(3.6);0.1	8.6(3.2);0.4	8.1(1.9);0.6	9.0(3.8);0.2	8.5(3.6);0.2	9.0(2.8);0.3

Positive Joint Work ANOVA

Power	Joint	Phase	F value	p value	DoF1	DoF2	0lbs	2.5 lbs	5 lbs	7.5 lbs	10 lbs	12.5 lbs	15 lbs	17.5 lbs	20 lbs	22.5 lbs	25 lbs	27.5 lbs	30 lbs
100W	Ankle	1	4.205	<0.001	2.225	11.124	3.6(3.5);1.4	3.6(3.5);1.4	3.6(3.5);1.4	4.6(2.5);1.3	4.9(4.2);1.2	5.1(3.6);1.2	8.9(8.0);0.7	6.0(4.9);0.9	5.0(4.0);1.2	6.1(5.6);0.9	7.1(4.1);0.8	7.2(7.2);0.7	7.3(8.1);0.6
100W	Ankle	2	4.363	<0.001	2.381	11.903	12.3(9.0);0.8	12.3(9.0);0.8	12.3(9.0);0.8	16.8(9.9);0.6	18.5(10.4);0.4	16.8(13.9);0.4	19.9(11.1);0.5	21.3(11.3);0.3	22.7(11.8);0.2	21.7(14.3);0.2	21.9(13.9);0.2	22.4(14.2);0.2	21.9(12.2);0.2
100W	Ankle	3	6.628	<0.001	1.611	8.054	2.2(1.7);1.6	2.2(1.7);1.6	2.2(1.7);1.6	1.3(1.1);1.4	1.0(0.7);1.5	1.0(1.2);1.0	1.4(2.2);0.8	0.3(0.3);0.8	1.3(2.5);0.7	0.4(0.4);0.7	0.8(0.8);1.0	0.7(0.8);0.9	2.0(3.1);0.8
100W	Ankle	4	1.961	0.034	2.873	14.363	1.3(1.2);0.4	1.3(1.2);0.4	1.3(1.2);0.4	0.5(0.3);0.2	0.4(0.5);0.4	1.2(2.4);0.2	0.7(0.7);0.0	0.4(0.3);0.2	0.5(0.8);0.2	0.5(0.5);0.2	0.5(0.4);0.3	0.5(0.9);0.2	0.4(0.4);0.3
100W	Knee	1	2.324	0.011	2.204	11.022	8.7(5.9);0.0	8.7(5.9);0.0	8.7(5.9);0.0	8.3(9.6);0.1	5.6(6.8);0.4	5.5(8.7);0.3	4.7(5.8);0.5	3.0(4.1);0.7	5.9(5.3);0.4	4.9(6.3);0.5	4.1(4.1);0.5	7.3(8.6);0.2	6.0(6.1);0.4
100W	Knee	2	0.585	0.868	2.122	10.611	4.5(6.0);0.1	4.5(6.0);0.1	4.5(6.0);0.1	5.7(9.0);0.1	5.3(7.2);0.1	4.8(5.9);0.1	5.5(4.3);0.0	5.0(6.6);0.0	4.8(7.6);0.0	2.8(2.9);0.5	3.3(3.1);0.4	4.4(4.2);0.1	7.2(11.8);0.2
100W	Knee	3	1.898	0.041	1.9	9.501	4.8(4.5);0.7	4.8(4.5);0.7	4.8(4.5);0.7	1.2(1.4);0.2	1.4(1.9);0.1	2.5(3.1);0.2	3.0(5.6);0.2	1.0(1.6);0.2	2.6(4.6);0.2	3.5(6.9);0.3	1.2(2.6);0.2	4.0(8.7);0.3	1.4(1.8);0.1
100W	Knee	4	1.196	0.298	3.081	15.407	4.9(2.6);0.0	4.9(2.6);0.0	4.9(2.6);0.0	3.7(2.6);0.3	3.3(1.5);0.5	3.2(2.6);0.5	3.1(2.4);0.7	2.8(1.8);0.6	3.0(0.9);0.7	2.7(1.1);0.8	4.1(2.9);0.2	2.9(2.0);0.6	3.0(2.2);0.6
100W	Hip	1	6.314	<0.001	2.749	13.746	83.1(47.1);0.9	83.1(47.1);0.9	83.1(47.1);0.9	102.5(75.3);0.5	116.4(65.4);0.4	100.3(68.7);0.6	127.6(58.5);0.4	145.0(59.2);0.2	120.6(49.5);0.4	118.1(61.2);0.4	127.4(54.9);0.3	123.5(56.9);0.3	121.6(40.5);0.4
100W	Hip	2	4.04	<0.001	2.359	11.795	70.3(48.3);0.5	70.3(48.3);0.5	70.3(48.3);0.5	70.9(48.1);0.5	81.4(64.8);0.3	99.6(61.7);0.0	121.9(81.1);0.1	95.5(60.5);0.2	116.9(92.7);0.2	107.9(87.3);0.1	103.6(70.6);0.0	105.1(60.4);0.0	114.0(60.1);0.2
100W	Hip	3	3.061	<0.001	2.141	10.703	95.7(31.4);0.5	95.7(31.4);0.5	95.7(31.4);0.5	98.7(31.4);0.6	93.4(30.9);0.4	83.7(29.1);0.1	87.5(35.2);0.2	94.6(32.7);0.3	79.4(18.6);0.1	82.5(33.0);0.1	89.8(33.6);0.3	87.0(34.3);0.3	84.8(34.2);0.2
100W	Hip	4	1.066	0.403	2.515	12.577	48.5(21.3);0.3	48.5(21.3);0.3	48.5(21.3);0.3	45.6(15.1);0.2	46.7(18.5);0.2	47.3(19.2);0.2	51.1(29.0);0.4	47.4(24.1);0.2	54.1(31.9);0.4	48.8(26.3);0.3	46.1(20.6);0.2	45.5(21.0);0.1	48.4(25.6);0.2
200W	Ankle	1	1.932	0.032	3.03	21.213	9.8(7.4);0.9	9.8(7.4);0.9	9.8(7.4);0.9	10.5(8.5);0.8	6.4(5.9);1.2	11.1(8.2);0.7	11.6(12.6);0.6	8.1(7.1);0.9	7.3(6.5);1.2	10.0(6.4);0.9	8.4(7.5);1.0	8.5(6.1);1.1	8.2(8.0);0.9
200W	Ankle	2	3.824	<0.001	1.952	13.662	30.5(16.9);0.5	30.5(16.9);0.5	30.5(16.9);0.5	26.8(17.7);0.6	26.0(14.2);0.5	33.5(24.3);0.3	36.2(19.5);0.2	34.9(25.6);0.3	37.4(22.0);0.2	38.3(19.4);0.2	34.8(23.5);0.3	38.7(20.2);0.1	28.1(15.5);0.8
200W	Ankle	3	4.199	<0.001	2.706	18.945	2.8(1.7);1.6	2.8(1.7);1.6	2.8(1.7);1.6	2.3(2.0);1.2	2.3(1.4);1.5	1.5(1.2);0.9	1.6(1.2);1.1	1.8(1.3);1.1	1.9(1.2);1.3	1.3(0.9);0.9	1.7(1.2);1.1	1.8(1.4);1.1	2.5(2.7);0.9
200W	Ankle	4	6.093	<0.001	3.229	22.604	1.7(1.0);0.5	1.7(1.0);0.5	1.7(1.0);0.5	1.3(0.8);0.2	1.2(1.0);0.1	1.1(1.5);0.1	0.6(0.4);0.4	1.1(1.7);0.3	0.5(0.4);0.4	0.7(0.6);0.3	0.7(0.5);0.3	0.7(0.5);0.3	1.2(1.4);0.5
200W	Knee	1	0.533	0.908	2.534	17.739	12.9(15.6);0.3	12.9(15.6);0.3	12.9(15.6);0.3	13.7(18.6);0.2	16.3(19.6);0.2	14.7(18.9);0.2	16.3(21.9);0.0	13.6(19.9);0.2	15.9(16.3);0.1	13.3(13.4);0.3	13.4(14.5);0.3	14.5(15.7);0.1	14.1(19.4);0.3
200W	Knee	2	1.708	0.066	3.497	24.479	5.2(6.9);0.2	5.2(6.9);0.2	5.2(6.9);0.2	5.5(6.0);0.2	4.1(3.8);0.3	7.1(8.1);0.0	6.9(5.6);0.1	5.8(5.5);0.4	7.7(8.5);0.1	6.0(5.7);0.2	8.9(10.0);0.2	10.5(15.9);0.2	10.3(13.5);0.2
200W	Knee	3	6.655	<0.001	2.583	18.083	8.4(5.4);1.1	8.4(5.4);1.1	8.4(5.4);1.1	5.2(5.0);0.5	4.0(3.7);0.3	3.5(3.2);0.2	5.4(5.4);0.4	4.9(4.1);0.9	3.2(4.6);0.1	3.6(4.0);0.2	3.7(4.0);0.2	3.7(3.0);0.2	3.5(3.3);0.7
200W	Knee	4	1.055	0.407	2.759	19.316	6.7(3.6);0.3	6.7(3.6);0.3	6.7(3.6);0.3	5.2(3.7);0.1	3.0(2.2);0.7	4.1(2.8);0.4	4.3(2.3);0.4	4.0(3.5);0.3	4.4(2.9);0.3	4.0(1.5);0.5	4.2(2.7);0.4	3.9(2.7);0.5	3.7(2.8);0.5
200W	Hip	1	6.309	<0.001	2.529	17.704	177.4(92.6);0.5	177.4(92.6);0.5	177.4(92.6);0.5	160.2(103.4);0.7	172.3(91.6);0.4	177.7(86.5);0.6	225.1(105.8);0.2	222.2(124.5);0.4	225.6(107.4);0.2	223.0(109.1);0.2	215.8(97.1);0.2	209.0(90.5);0.4	192.5(108.4);0.5
200W	Hip	2	2.533	0.004	2.901	20.309	139.3(78.6);0.4	139.3(78.6);0.4	139.3(78.6);0.4	98.0(46.5);0.8	143.2(68.6);0.1	157.2(86.4);0.2	176.2(119.0);0.1	178.8(124.1);0.1	163.5(109.0);0.1	160.9(103.5);0.2	153.6(103.2);0.2	176.1(97.8);0.1	125.5(76.0);0.6
200W	Hip	3	2.557	0.004	2.047	14.326	102.9(26.8);0.7	102.9(26.8);0.7	102.9(26.8);0.7	94.2(35.3);0.5	89.2(38.5);0.4	90.9(27.8);0.4	100.5(36.2);0.5	97.6(34.8);0.3	96.4(30.0);0.6	93.6(28.7);0.5	94.1(30.8);0.5	94.4(29.2);0.4	88.3(34.5);0.2
200W	Hip	4	2.051	0.021	2.409	16.862	56.7(22.0);0.6	56.7(22.0);0.6	56.7(22.0);0.6	50.0(17.5);0.4	53.6(26.8);0.4	54.1(18.6);0.6	51.1(19.3);0.4	56.1(21.1);0.6	50.3(18.5);0.4	48.7(19.9);0.3	45.6(23.5);0.2	57.1(23.7);0.6	48.9(24.4);0.3

Negative Joint Work ANOVA

Power	Joint	Phase	F value	p value	DoF1	DoF2	0lbs	2.5 lbs	5 lbs	7.5 lbs	10 lbs	12.5 lbs	15 lbs	17.5 lbs	20 lbs	22.5 lbs	25 lbs	27.5 lbs	30 lbs
100W	Ankle	1	1.214	0.286	1.53	7.65	-2.1(1.7);0.5	-2.1(1.7);0.5	-2.1(1.7);0.5	-1.6(1.6);0.2	-1.2(1.1);0.0	-1.3(1.2);0.1	-1.1(1.1);0.1	-1.0(1.2);0.1	-1.8(2.2);0.3	-1.6(2.2);0.2	-1.4(1.7);0.1	-1.8(1.7);0.3	-2.0(2.5);0.4
100W	Ankle	2	0.394	0.972	1.991	9.957	-3.4(1.7);0.2	-3.4(1.7);0.2	-3.4(1.7);0.2	-3.5(3.2);0.1	-3.1(2.6);0.2	-4.6(4.1);0.0	-3.7(2.4);0.2	-2.8(1.9);0.0	-3.1(4.2);0.2	-2.9(3.0);0.2	-2.8(1.9);0.3	-3.1(4.2);0.2	-3.5(3.7);0.1
100W	Ankle	3	5.161	<0.001	1.896	9.479	-1.3(1.1);1.8	-1.3(1.1);1.8	-1.3(1.1);1.8	-1.8(1.3);1.6	-2.5(1.9);1.3	-2.4(1.7);1.3	-4.5(3.6);0.9	-3.9(3.1);0.6	-5.0(5.7);0.3	-4.0(2.7);0.8	-2.9(1.5);1.1	-4.5(3.3);0.6	-4.5(3.9);0.5
100W	Ankle	4	2.03	0.027	1.706	8.531	-0.6(0.8);1.0	-0.6(0.8);1.0	-0.6(0.8);1.0	-1.0(0.8);0.8	-1.3(1.3);0.6	-1.0(1.3);0.6	-1.8(1.4);0.5	-1.2(0.9);0.6	-1.9(1.7);0.3	-1.8(1.9);0.3	-1.6(1.3);0.4	-1.8(1.8);0.4	-2.4(2.4);0.1
100W	Knee	1	1.26	0.254	3.971	19.853	-57.8(36.2);0.0	-57.8(36.2);0.0	-57.8(36.2);0.0	-54.7(42.6);0.1	-58.5(41.5);0.0	-53.2(33.3);0.2	-62.6(37.6);0.1	-62.5(40.7);0.0	-47.4(30.2);0.2	-47.8(33.9);0.2	-52.3(31.2);0.2	-46.2(35.8);0.2	-50.0(37.6);0.2
100W	Knee	2	2.802	0.002	3.295	16.473	-58.6(34.0);0.2	-58.6(34.0);0.2	-58.6(34.0);0.2	-61.9(38.0);0.1	-66.4(42.6);0.0	-83.6(49.4);0.3	-81.9(53.0);0.1	-72.0(44.5);0.2	-75.6(55.7);0.1	-86.4(65.4);0.3	-73.3(47.6);0.0	-74.5(43.0);0.1	-66.0(37.1);0.1
100W	Knee	3	10.721	<0.001	3.554	17.769	-38.0(18.3);1.8	-38.0(18.3);1.8	-38.0(18.3);1.8	-56.6(21.8);1.0	-58.5(24.0);0.9	-64.9(28.8);0.8	-66.7(20.0);0.7	-72.2(26.7);0.6	-67.5(23.2);0.6	-66.6(27.0);0.6	-82.0(28.0);0.1	-70.7(34.7);0.4	-74.4(28.9);0.3
100W	Knee	4	12.24	<0.001	2.142	10.708	-10.3(16.0);1.8	-10.3(16.0);1.8	-10.3(16.0);1.8	-20.3(12.8);1.3	-25.9(14.4);0.9	-34.4(20.1);0.2	-35.8(22.4);0.2	-32.7(19.7);0.3	-39.0(24.5);0.0	-36.2(24.3);0.1	-35.2(22.4);0.1	-35.0(18.8);0.2	-36.4(18.3);0.1
100W	Hip	1	0.794	0.672	2.009	10.046	-1.6(3.0);0.2	-1.6(3.0);0.2	-1.6(3.0);0.2	-4.7(10.9);0.4	-1.5(2.4);0.2	-8.2(15.9);0.6	-3.0(3.4);0.7	-1.4(1.8);0.3	-2.7(2.5);0.8	-3.8(7.3);0.5	-1.6(1.8);0.3	-1.9(2.3);0.4	-2.6(3.2);0.6
100W	Hip	2	4.005	<0.001	1.751	8.755	-6.3(5.5);0.8	-6.3(5.5);0.8	-6.3(5.5);0.8	-5.8(4.0);0.8	-4.0(2.8);0.5	-4.2(4.2);0.3	-3.2(2.9);0.3	-3.4(4.0);0.3	-4.0(5.6);0.3	-3.0(2.7);0.1	-3.3(3.4);0.2	-3.8(3.8);0.3	-5.5(8.1);0.4
100W	Hip	3	1.236	0.27	1.243	6.214	-2.6(8.7);0.2	-2.6(8.7);0.2	-2.6(8.7);0.2	-1.5(4.1);0.0	-1.3(3.9);0.0	-2.4(3.7);0.2	-1.9(3.6);0.0	-0.4(0.6);0.1	-1.5(2.8);0.0	-1.7(4.1);0.0	-0.3(0.6);0.4	-1.1(2.0);0.1	-2.0(3.4);0.1
100W	Hip	4	10.632	<0.001	3.531	17.653	-0.6(0.7);1.8	-0.6(0.7);1.8	-0.6(0.7);1.8	-3.4(4.5);0.9	-4.1(4.2);0.8	-6.2(4.8);0.5	-5.8(3.4);0.8	-5.6(3.3);0.7	-5.4(4.4);0.5	-6.6(6.3);0.3	-5.8(4.4);0.5	-7.2(7.3);0.2	-6.9(4.7);0.3
200W	Ankle	1	1.168	0.312	3.286	23.005	-3.3(2.7);0.1	-3.3(2.7);0.1	-3.3(2.7);0.1	-2.4(2.6);0.1	-3.8(3.6);0.3	-3.2(3.3);0.1	-3.9(4.2);0.2	-4.4(4.3);0.6	-5.1(6.6);0.4	-3.8(4.9);0.2	-3.8(3.6);0.3	-3.9(3.1);0.2	-3.3(2.9);0.3
200W	Ankle	2	0.515	0.919	2.015	14.108	-3.4(5.4);0.1	-3.4(5.4);0.1	-3.4(5.4);0.1	-4.6(6.6);0.1	-4.5(4.6);0.0	-5.1(6.9);0.1	-3.5(4.5);0.1	-4.0(6.8);0.3	-3.2(5.3);0.1	-3.1(5.4);0.1	-5.0(9.4);0.1	-4.8(11.7);0.0	-3.9(5.2);0.4
200W	Ankle	3	4.592	<0.001	2.543	17.799	-1.1(0.9);1.5	-1.1(0.9);1.5	-1.1(0.9);1.5	-2.1(2.0);1.0	-1.3(1.6);1.2	-2.0(1.3);1.2	-1.6(1.8);1.1	-1.7(1.3);1.1	-1.6(1.4);1.3	-1.9(1.5);1.2	-1.7(1.9);1.2	-1.6(1.6);1.1	-1.4(1.8);1.2
200W	Ankle	4	6.023	<0.001	2.227	15.589	-0.6(0.6);1.1	-0.6(0.6);1.1	-0.6(0.6);1.1	-0.6(0.4);1.1	-0.8(0.9);0.9	-0.8(0.7);1.0	-1.2(1.1);0.8	-0.9(1.1);1.1	-1.2(0.9);0.9	-1.5(1.4);0.7	-1.2(0.8);0.9	-1.3(1.3);0.7	-1.3(1.1);0.9
200W	Knee	1	1.016	0.444	2.635	18.442	-74.4(65.1);0.1	-74.4(65.1);0.1	-74.4(65.1);0.1	-62.5(51.9);0.0	-61.1(55.1);0.2	-65.7(55.5);0.0	-75.6(62.6);0.1	-75.8(58.1);0.1	-69.2(56.8);0.1	-67.8(57.8);0.0	-71.1(57.5);0.1	-62.3(46.8);0.1	-72.0(54.8);0.1
200W	Knee	2	1.165	0.314	3.426	23.983	-89.5(58.8);0.1	-89.5(58.8);0.1	-89.5(58.8);0.1	-90.6(55.3);0.1	-97.5(59.5);0.2	-102.2(61.1);0.0	-102.6(79.8);0.0	-120.9(81.0);0.3	-99.9(76.3);0.0	-99.9(74.7);0.0	-93.9(66.9);0.1	-97.2(62.1);0.1	-76.8(44.5);0.2
200W	Knee	3	5.972	<0.001	3.023	21.163	-35.9(19.2);1.0	-35.9(19.2);1.0	-35.9(19.2);1.0	-51.7(25.8);0.5	-40.2(13.1);0.8	-52.0(28.1);0.5	-53.1(30.1);0.5	-48.5(27.9);0.6	-53.1(25.7);0.5	-51.3(27.4);0.5	-53.1(25.7);0.5	-49.0(30.0);0.6	-49.1(22.9);0.5
200W	Knee	4	8.634	<0.001	3.059	21.413	-9.6(10.0);1.4	-9.6(10.0);1.4	-9.6(10.0);1.4	-22.8(18.4);0.5	-26.2(21.6);0.2	-28.0(20.7);0.2	-27.2(22.0);0.2	-31.6(26.7);0.0	-25.7(19.8);0.3	-25.4(21.2);0.3	-24.1(20.5);0.4	-28.8(21.2);0.1	-23.7(21.3);0.4
200W	Hip	1	1.334	0.202	2.321	16.248	-1.2(1.2);0.4	-1.2(1.2);0.4	-1.2(1.2);0.4	-3.5(5.4);0.3	-3.6(5.8);0.3	-2.8(3.4);0.3	-2.1(2.7);0.2	-3.2(3.1);0.3	-1.9(2.4);0.0	-1.7(2.0);0.1	-1.1(1.1);0.4	-2.5(2.3);0.3	-3.6(5.3);0.3
200W	Hip	2	1.769	0.054	2.62	18.343	-2.1(2.1);0.7	-2.1(2.1);0.7	-2.1(2.1);0.7	-9.0(21.8);0.5	-3.5(8.4);0.4	-3.2(6.2);0.5	-3.1(6.3);0.5	-1.8(2.0);0.4	-5.7(13.7);0.5	-3.0(5.9);0.5	-4.9(12.6);0.4	-5.4(11.8);0.5	-8.5(17.5);0.6
200W	Hip	3	0.701	0.769	1.516	10.615	-0.9(2.3);0.1	-0.9(2.3);0.1	-0.9(2.3);0.1	-2.7(6.2);0.3	-2.0(5.1);0.1	-1.7(4.5);0.1	-1.3(3.9);0.0	-1.2(2.9);0.3	-2.1(6.8);0.2	-1.1(3.2);0.0	-1.7(4.6);0.1	-1.8(4.8);0.1	-3.7(7.4);0.6
200W	Hip	4	6.72	<0.001	2.08	14.558	-1.9(1.9);1.2	-1.9(1.9);1.2	-1.9(1.9);1.2	-6.1(7.6);0.4	-8.2(9.0);0.1	-5.8(4.4);0.6	-7.0(5.7);0.4	-7.0(5.1);0.6	-8.4(9.5);0.1	-8.0(6.1);0.2	-9.5(10.4);0.0	-7.3(7.9);0.3	-8.3(7.2);0.2
