# Supplementary figures and images for: Counterweight mass influences single-leg cycling biomechanics
Source: PLoS One. 2024 Jun 7;19(6):e0304136. doi: 10.1371/journal.pone.0304136 (PMC11161077; doi:10.1371/journal.pone.0304136)

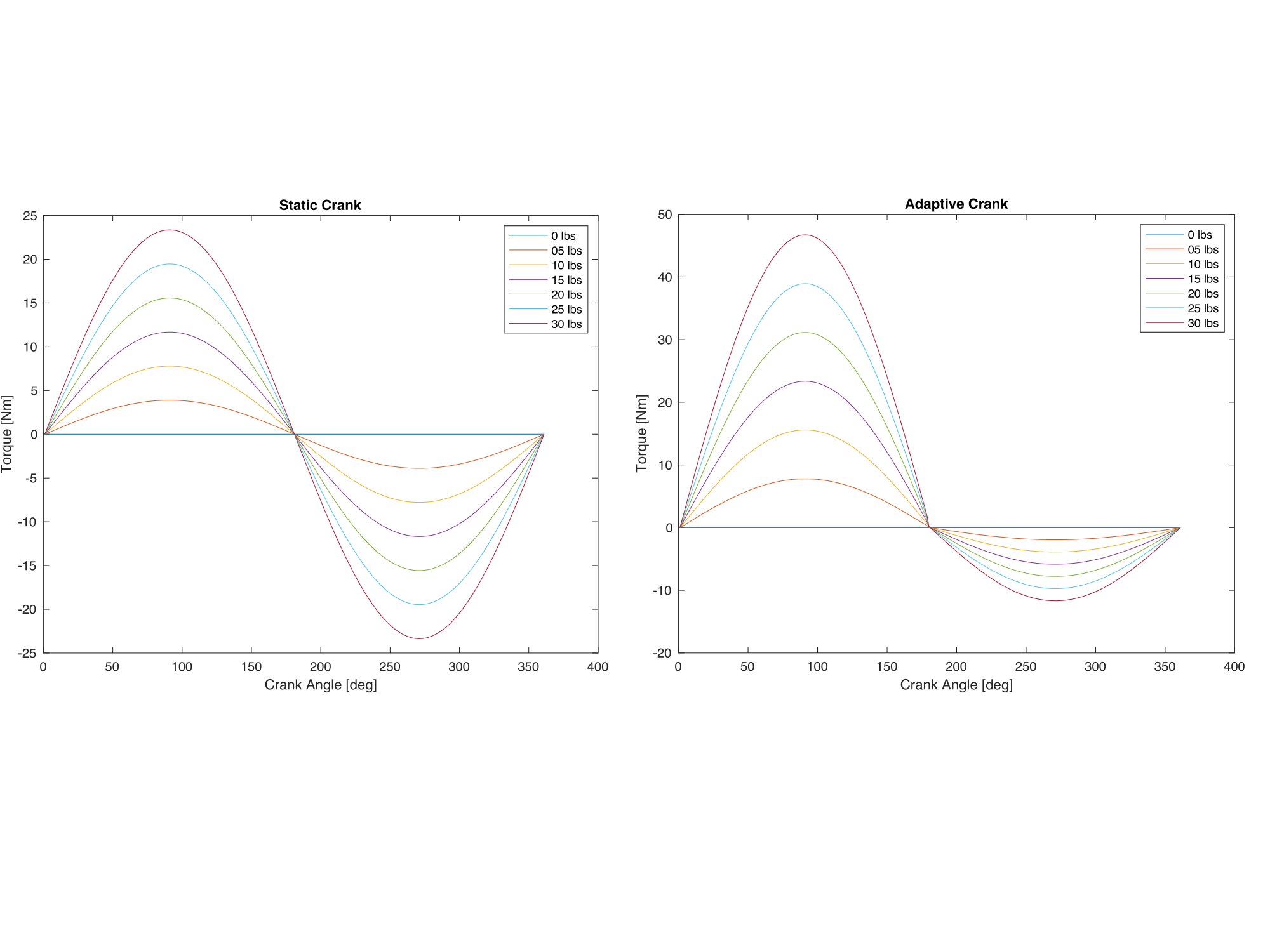

Supplement: S2 Fig — The static crank data (top figure) models the effects of the different counterweights used in this study on crank torque depending on the different crank angle and given a constant crank length. The adaptive crank data models the effects of the different counterweights used in this study on crank torque depending on the different crank angle and given a changing crank length that increases by an order of 2 on the downstroke and decrease by an order of 2 during the upstroke. (TIFF) [file pone.0304136.s002.tiff]

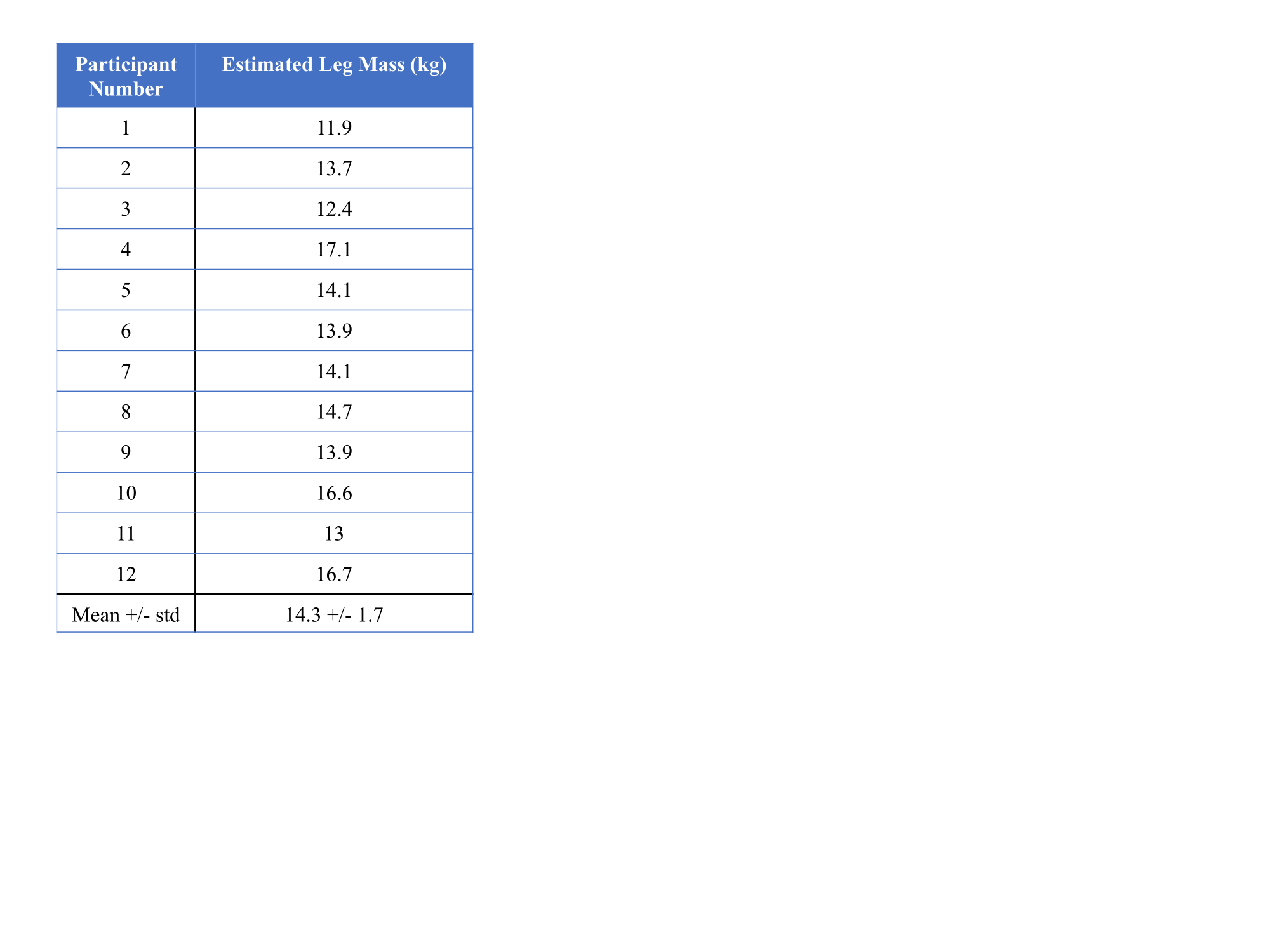

Supplement: S1 Table — The table represents the estimated mass of the leg for each participant as well as the group mean and standard deviation. (TIFF) [file pone.0304136.s003.tiff]
