# Supplementary material for: Counterweight mass influences single-leg cycling biomechanics
Source: PLoS One. 2024 Jun 7;19(6):e0304136. doi: 10.1371/journal.pone.0304136 (PMC11161077; doi:10.1371/journal.pone.0304136)
Supplement: S1 File — The 2-way ANOVA for counterweight mass and power output for single-leg cycling (i.e., 50W, 100W). The single-leg cycling data which was normalized by the double-leg cycling data for each variable of interest. (PDF) [file pone.0304136.s004.pdf]

Relative Changes Between Different Counterweight (Mass) Conditions

Joint Angles

Phase 1

The joint angle analysis indicated that there were significant differences in peak-to-peak knee joint angles ($F_{(12,60)}$ = 2.240, p = 0.021), but not the knee, or hip joints. The post hoc analysis did not indicate any relevant significant differences.

Phase 2

The joint angle analysis indicated that there were no significant differences in peak-to-peak ankle, knee, or hip joint.

Phase 3

There were no significant differences for peak to peak angles for the ankle and knee joints, but there were significant differences for the hip joint ($F_{(12,60)}$ = 2.553, p = 0.009). The post hoc analysis did not indicate any relevant significant differences.

Phase 4

There were no significant differences for peak to peak angles for the ankle and knee joints, but there were significant differences for the hip joint ($F_{(12,60)}$ = 2.491, p = 0.010). The post hoc analysis did not indicate any relevant significant differences.

Pedal Forces

Phase 1

The peak-to-peak pedal forces tangential to the crank produced a significant interaction effect ($F_{(12,84)}$ = 2.955, p = 0.002) and the post hoc analysis indicated differences between the 0, 2.5, 5,

and 7.5 lbs conditions. The peak-to-peak pedal forces parallel to the crank were not significantly different, but the forces in line with the axis of rotation showed a significant interaction effect ($F_{(12,84)} = 4.923$, $p < 0.001$), but the post hoc analysis did not reveal any significant differences between the different conditions. The mean pedal forces tangential to the crank produced a significant interaction effect ($F_{(12,84)} = 14.349$, $p < 0.001$) and the post hoc analysis indicated differences between the 0, 2.5, 5, and 7.5 lbs conditions. The peak-to-peak pedal forces parallel to the crank and in line with the axis of rotation of the crank were not significantly different.

Phase 2

The peak-to-peak pedal forces tangential to the crank produced a significant interaction effect ($F_{(12,84)} = 2.003$, $p = 0.034$) and the post hoc analysis indicated differences between the 0, 5, and 7.5 lbs conditions. The peak-to-peak pedal forces parallel to the crank showed a significant main effect between the different power outputs ($F_{(1,7)} = 9.258$, $p = 0.019$) and the post hoc analysis indicated differences between the 20 and 25 lbs conditions. The peak-to-peak pedal forces in line with the axis of rotation of the crank showed a significant interaction effect ($F_{(12,84)} = 3.281$, $p < 0.001$) and the post hoc analysis did not reveal any significant differences across conditions. The mean pedal forces tangential to the crank produced a significant interaction effect ($F_{(12,84)} = 6.382$, $p < 0.001$) and the post hoc analysis indicated differences between the 0, 2.5, and 5 lbs conditions. The mean pedal forces parallel to the crank showed a significant interaction effect ($F_{(12,84)} = 3.814$, $p < 0.001$) and the post hoc analysis indicated differences between the 5 and 10 lbs conditions. The mean pedal forces in line with the axis of rotation of the crank were not significantly different.

Phase 3

The peak-to-peak pedal forces tangential to the crank were significantly different ($F_{(12,84)}$ = 11.101, $p < 0.001$) and the post hoc analysis indicated differences between the 0 lbs condition. The peak-to-peak pedal forces parallel to the crank showed a significant interaction effect ($F_{(12,84)}$ =5.190, $p < 0.001$) and the post hoc analysis did not show any differences across the conditions. The peak-to-peak pedal forces in line with the axis of rotation of the crank showed a significant interaction effect ($F_{(12,84)}$ = 2.070, $p = 0.028$) and the post hoc analysis revealed significant differences between the 0 lbs condition. The mean pedal forces tangential to the crank produced a significant main effect ($F_{(1,7)}$ = 6.083, $p = 0.043$) and the post hoc analysis indicated differences between the 20 lbs condition. The mean pedal forces parallel to the crank showed a significant interaction effect ($F_{(12,84)}$ =11.014, $p < 0.001$) and the post hoc analysis showed significant differences between the 20, 22.5, 25, and 27.5 lbs condition. The mean pedal forces in line with the axis of rotation of the crank were not significantly different.

Phase 4

The peak-to-peak pedal forces tangential to the crank were significantly different ($F_{(12,84)}$ = 11.882, $p < 0.001$) and the post hoc analysis indicated differences between the 0 lbs condition. The peak-to-peak pedal forces parallel to the crank showed a significant interaction effect ($F_{(12,84)}$ =2.146, $p = 0.022$) and the post hoc analysis indicated differences between the 12.5, 15, 20, 22.5, 25, and 27.5 lbs conditions. There were no significant differences in the peak to peak forces in line with the axis of rotation. The mean pedal forces tangential to the crank produced a significant main effect ($F_{(1,7)}$ = 6.156, $p = 0.042$), but the post hoc analysis indicated no significant differences. The mean pedal forces parallel to the crank and in line with the axis of rotation showed no significant differences.

Joint Moments

Phase 1

The mean joint moment at the ankle showed a significant interaction effect ($F_{(12,60)}$ =2.856, p = 0.004) with significant differences between the 0, 2.5, and 5 lbs conditions. There were no significant differences in mean knee and hip joint moments and peak to peak moments at the ankle, knee, and hip between the power outputs.

Phase 2

The mean joint moment at the ankle showed a significant interaction effect ($F_{(12,60)}$ =3.052, p = 0.002) with significant differences between the 0 lbs condition. The mean joint moment at the knee showed a significant interaction effect ($F_{(12,60)}$ =2.074, p = 0.033), but the post hoc analysis did not reveal any significant differences. There were no significant differences in the mean hip joint moment and peak to peak moments at the ankle, knee, and hip.

Phase 3

There were no significant differences in mean and peak to peak ankle, knee, and hip joint moments between the power outputs.

Phase 4

There was a significant interaction effect at the knee ($F_{(1,7)}$ =4.176, $p < 0.001$) and hip ($F_{(1,7)}$ =5.288, $p < 0.001$) for peak to peak moments, but the post hoc analysis did not reveal any significant differences. There were was a significant main effect for different power outputs at the hip joint ($F_{(1,7)}$ =6.871, p = 0.047), but no differences as a result of the post hoc analysis. There were no significant differences in mean ankle and knee joint moments between the power outputs.

Joint Work

Phase 1

There were no significant differences in positive or negative work at the ankle, knee, and hip joints.

Phase 2

There were no significant differences in positive or negative work at the ankle and knee joints. There were no differences in negative hip work, but there were significant differences in positive joint work at the hip ($F_{(12,60)}$ =2.142, p = 0.027) and the post hoc analysis indicated that there were significant differences for the 30 lbs condition.

Phase 3

There were no significant differences in positive or negative work at the ankle and knee joint. There were no differences in negative hip work, but there were significant differences in positive joint work at the hip ($F_{(12,60)}$ =2.153, p = 0.026), yet there were no significant differences as a result of the post hoc test.

Phase 4

There were no significant differences in positive work at the ankle and knee joint. There was a significant interaction effect for negative work at the ankle joint ($F_{(12,60)}$ =2.062, p = 0.034), but there were no significant differences as a result of the post hoc test. There was also significant interaction effect for negative work at the knee joint ($F_{(12,60)}$ =2.444, p = 0.012) and the post hoc analysis indicated that there were significant differences for the 30 lbs condition. There were no significant differences in positive or negative hip work.
